# Impact assessment of effective rounds of mass drug administration against schistosomiasis and soil transmitted helminthiasis among school age children in Akwa Ibom State, Nigeria

**DOI:** 10.1371/journal.pntd.0014603

**Published:** 2026-08-07

**Authors:** Kenneth Nnamdi Opara, Clement Ameh Yaro, Nnamso Williams Ikpeida, Aliyu Mohammed, Hadiza Joy Marcus, Kingsley Azu, Salisu Ahmad, Esther Sunday Okon, Ellie Leaning, Moses Aderogba, Louise-Makau Barasa, Antia Etop, Ime Sylvester Udoh, Fatai Oyediran, Imabong O. Umah, Solomon M. Jacob, Gideon U. Ntuen, Veronica S. Augustine, Halima Toro

**Affiliations:** 1 Department of Animal and Environmental Biology, Faculty of Biological Sciences, University of Uyo, Uyo, Akwa Ibom, Nigeria; 2 Helen Keller International, Abuja, Nigeria; 3 The END Fund, New York, USA; 4 The END Fund, Abuja, Nigeria; 5 Akwa Ibom State Ministry of Health, Uyo, Akwa Ibom, Nigeria; 6 Federal Ministry of Health, Abuja, Nigeria; Washington University School of Medicine, UNITED STATES OF AMERICA

## Abstract

**Background:**

Schistosomiasis (SCH) and Soil-transmitted helminthiasis (STH) remain major neglected tropical diseases across sub-Saharan Africa. In Akwa Ibom State, preventive chemotherapy (PC) has been implemented in endemic Local Government Areas (LGAs). This study assessed the impact of five effective rounds of PC on the prevalence and intensity of SCH and STH in 26 endemic LGAs.

**Methods:**

This study was carried out in 26 LGAs of Akwa Ibom State between September and October 2025. A school-based cross-sectional survey was employed. Schools were purposefully selected from endemic wards, and a total of 200–300 samples were collected per LGA from school children of both sexes aged 8–14 years. Samples were collected using a systematic random sampling method. Questionnaires were administered using electronic data forms. Stool samples collected were analyzed using the Kato-Katz technique, while urine samples were analyzed using the urine filtration method. Conventional microscopy alongside a novel AiDx digital microscope was used to examine prepared specimen. Data obtained were analyzed using descriptive statistics, chi-square test, bivariate and multivariate logistic regression on SPSS, and maps were generated using ArcMap.

**Results:**

No cases of *Schistosoma mansoni* or *Schistosoma haematobium* were detected across the 26 LGAs (0.0% prevalence). The overall prevalence of STH was 27.62% (95% CI: 26.54–28.72), ranging from 4.92% to 71.65% across LGAs. Species-specific prevalence was 15.38% (95% CI: 14.52–16.28, range: 3.3% to 38.3%) for hookworm, 15.10% (95% CI: 14.24–15.99, range: 1.6% to 67.0%) for *Ascaris lumbricoides*, and 1.52% (95% CI: 1.25–1.85, range: 0.4% to 9.3%) for *Trichuris trichiura*. Mbo (71.65%) and Udong Uko (62.04%) recorded the highest prevalences, whereas Ika (4.92%) had the lowest. Prevalence was significantly higher among children aged 11–14 years (31.77%; 95% CI: 29.95–33.64) compared with those aged 8–10 years (95% CI: 23.77–26.46), and among males (29.30%; 95% CI: 27.80–30.86) compared with females (25.73%; 95% CI: 24.24–27.35) (p < 0.05). 98.66%, 99.09% and 100.0% of schoolchildren had light infections of *A. lumbricoides*, hookworms and *T. trichiura* respectively. Compared with baseline data, STH prevalence declined by 30.15%. Significant risk factors associated with STH include; males (aOR = 1.196, 95% CI: 1.072 – 1.335), age group of 11–14 years (aOR = 1.391, 95% C.I.: 1.244 – 1.554), dry pit latrine (aOR = 1.464, 95% C.I. = 1.239 – 1.731), schools where children urinate (aOR = 2.620, 95% C.I.: 2.362 – 2.929) and defecate (aOR = 1.238, 95% C.I. = 1.097 – 1.396) around school or wait to defecate after school (aOR = 1.830, 95% C.I. = 1.365 – 2.454), schoolchildren that drinks from surface water (aOR = 1.731, 95% C.I. = 1.486 – 2.017) or boreholes that uses hand pump (aOR = 1.666, 95% C.I. = 1.412 – 1.965).

**Conclusion:**

Schistosomiasis was not reported in 26 LGAs of Akwa Ibom. Soil-transmitted helminths had a significant reduction in prevalence, but with varying prevalence across the states. There is a need for sustained preventive chemotherapy against STH in the State. Improvement in water, sanitation, and hygiene (WASH) infrastructures to create behavioural change, and strengthen the control efforts.

## Author summary

This study assessed the impact of 5 rounds of mass drug administration (MDA) on schistosomiasis and soil-transmitted helminthiasis (STH) among 6,431 school-aged children. In this current study, no cases of schistosomiasis were detected in school children in 26 LGAs. STH prevalence dropped by 30.15% to 27.62%, but disparities persist across regions (4.92% to 71.65%). Hookworm and *Ascaris lumbricoides* are the main STH species. Risk factors for STH include age 11–14 years, male, poor sanitation and inadequate toilet facilities. The MDA effort succeeded in eliminating schistosomiasis, but sustained interventions are required for STH, along with improved WASH infrastructures and behavioural changes to meet the WHO 2030 targets.

## 1 Introduction

Schistosomiasis (SCH) and soil-transmitted helminthiasis (STH) are among the most prevalent neglected tropical diseases (NTDs), disproportionately affecting impoverished communities in sub-Saharan Africa [1,2]. SCH, caused by *Schistosoma* species (primarily *S. haematobium* in Nigeria), leads to urinary tract damage, haematuria, anaemia, and increased HIV susceptibility, with over 240 million global cases [3]. STH, including *Ascaris lumbricoides*, *Trichuris trichiura,* and hookworms, causes malnutrition, growth stunting, and cognitive impairment, infecting 1.5 billion people worldwide [4].

In terms of burden, Nigeria has been at the forefront of the African region, with 134 million people at risk of SCH and widespread STH transmission [5]. The WHO NTD Roadmap 2021–2030 targets both diseases for elimination as a public health problem (EPHP) for SCH (<1% prevalence in School-Age Children (SAC)) and STH (<2% moderate/heavy intensity) by 2030, with impact assessments to be done after 3–5 MDA rounds [6].

Prior to the impact assessment, the preventive chemotherapy (PC) strategy implemented in Akwa Ibom State was led by the Federal Ministry of Health (FMoH) in collaboration with the State Ministry of Health and supported by Helen Keller Intl focused on large scale Mass Administration of Medicines (MAM) targeting school-aged children (SAC) aged 5–14 years. For STH, the strategy mandated biannual PC in areas with prevalence above 50%, and annual PC in regions with a 20% to 49.9% prevalence with over 75% coverage across implementation units (IUs) using albendazole or menbendazole, while SCH control among SAC relied on a case-management approach utilizing praziquantel [7]. Earlier baseline studies in Akwa Ibom State covered all the 31 LGAs. The baseline surveys (2013–2015) in Akwa Ibom State revealed a high level of STH endemicity (25.1 - 91.4%), and a low level of SCH prevalence (0.0 - 1.2%) [8,9]. In 2024, an impact assessment survey was carried out in five (5) LGAs that has achieved five effective rounds of mass drug administration (MDA). The current assessment targets the remaining 26 LGAs that have similarly completed five effective treatment rounds, with the aim of evaluating the impact of the intervention and informing decisions on the next phase of the elimination programme.

This study evaluates the impact of PC on the prevalence, intensity and endemicity of SCH and STH across 26 LGAs of Akwa Ibom State. The assessment follows five effective rounds of PC and is designed to determine whether the elimination as a public health problem (EPHP) targets have been achieved in accordance with the WHO monitoring and evaluation framework [7]. The survey is part of the national and state NTD strategic plan to provide data needed for planning, implementation, and monitoring the progress of the SCH and STH control programme in Akwa Ibom State.

## 2 Methods

### 2.1 *Ethical considerations*

The Akwa Ibom State Research Ethics Committee provided ethical approval (AKHREC/08/09/25/376). Community gatekeepers were provided with administrative clearance and letters of introduction. Parents or guardians of participating school-aged children (SAC) provided written informed consent and assent of children was obtained where necessary. It was voluntary and confidential.

### 2.2 *Study site*

**Fig 1:**
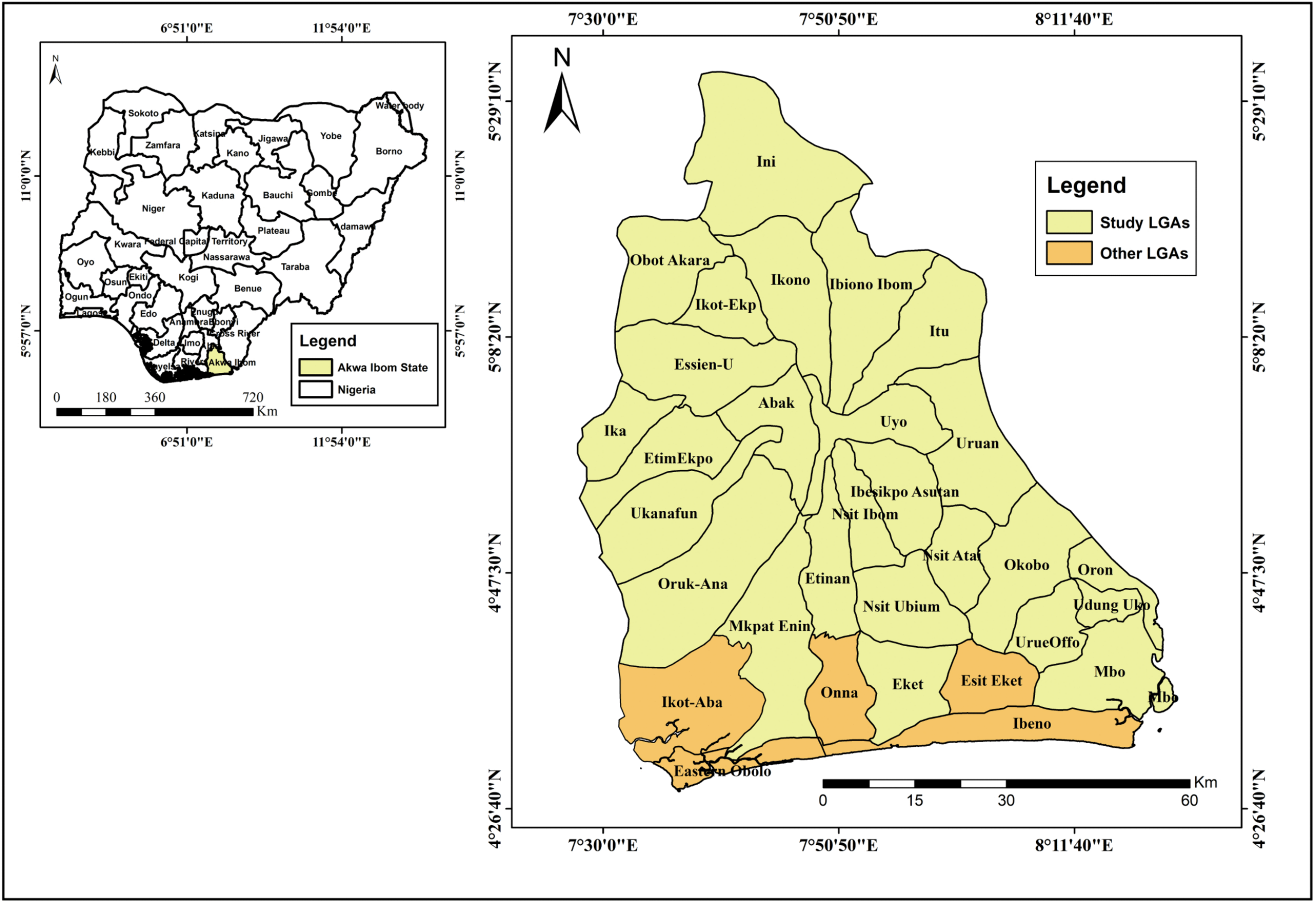
Map of the study area indicating LGAs surveyed. This figure was created by the authors on ArcMap 10.8 software. Available at https://desktop.arcgis.com/en/arcmap/index.html. The Nigeria and Akwa Ibom State shapefile was obtained from https://data.humdata.org/dataset/geoboundaries-admin-boundaries-for-nigeria, a publicly accessible database and are licensed under CC BY 4.0 (https://data.humdata.org). The authors allow unrestricted reuse of this map provided that proper credit is given to the source.

Akwa Ibom State is situated in the south-south region of Nigeria. Its location is between latitudes 4°32’1’’ and 5°33’1’’ North and longitudes 7°25’1’’ and 8°25’1’’ East. The state shares its borders with Rivers State on the East side, Cross River on the West, Abia State on the North and the Gulf of Guinea on the South. The study was conducted across 26 selected LGAs of Akwa Ibom State. These LGAs includes; Abak, Eket, Essien Udim, Etim Ekpo, Etinan, Ibesikpo Asutan, Ibiono Ibom, Ika, Ikono, Ikot Ekpene, Ini, Itu, Mbo, Mkpat Enin, Nsit Atai, Nsit-Ibom, Nsit Ubium, Obot Akara, Okobo, Oron, Oruk Anam, Udung Uko, Ukanafun, Uruan, Urue-Offiong/Oruko and Uyo (Fig 1).

### 2.3 *Community sensitization/mobilization*

The community mobilization was done by advocacy visits, local media and working with school authorities and community leaders. The research objectives, methods and voluntary nature of the study were clearly explained before the data collection.

### 2.4 *Study design*

A cross-sectional impact assessment among school-aged children was undertaken in October 2025 after the implementation of 5–8 effective rounds of mass drug administration (MDA). The study employed a two-stage sampling design aligned with the WHO [7] guidelines for impact assessment. Initially, five (5) wards were purposively selected from each Local Government Area (LGA), taking into account their epidemiological importance and baseline endemicity profiles identified during the baseline survey.

#### 2.4.1 *Selection of primary sampling units (PSUs).*

In each implementation unit (IU), the school with the highest prevalence of STH and SCH was purposively selected as the primary sampling unit (PSU). The selection was made from the list of eligible schools within the IU resulting in a total of five schools per LGA and 130 PSUs in 26 LGAs.

#### 2.4.2 *Selection of participants.*

A systematic sampling method was used to randomly select 52 SAC aged 8 years to 14 years (26 males and 26 females) in every PSU [7,10]. In cases that the enrolment was more than the target numbers, random starting point and sample interval were used. In accordance with this criterion the total sample size of over 7,000 was aimed to be reached. There were however 6,431 valid samples that were analysed.

### 2.5 *Sample collection*

School-aged children were grouped in terms of gender and age by a systematic random sampling procedure. Sampling intervals were applied in the situations where the enrolment was larger than the required (e.g., SI = total SAC/required; every n^th^ child sampled at a random starting point). All chosen children gave stool sample (Collected in labelled containers) and urine sample collected mid-morning (10:00–14:00 hours) so as to maximise the detection of *Schistosoma haematobium* egg excretion.

### 2.6 *Laboratory examination of samples*

Samples collected were transported to the laboratory for processing and examination. Parasite eggs in stools were analysed with the use of the Kato-Katz method on STH (Ascaris lumbricoides, *Trichuris trichiura*, and hookworm) and intestinal schistosome (*Schistosoma mansoni*) [1,11]. Hookworm slides were read in less than 60 minutes to avoid the clearance of eggs. The counts of eggs were converted in terms of eggs per gram (epg) and categorized based on WHO intensity thresholds. Urine was screened for haematuria using Combi-9 reagent strips, followed by filtration using filtration kits, and thereafter microscopy for *S. haematobium* eggs. Conventional microscope was used to examine prepared slides.

For quality control, 10% of slides were re-read by a senior parasitologist and blinded duplicate Kato-Katz was carried out on 20% of samples [12]. The AiDx NTDx device, a digital microscope with performance accuracy of 95% was used alongside conventional microscope to increase quality control during sample examination [13,14]. Intensity was classified according to WHO standards.

### 2.7 *Electronic data collection*

The electronic data collection was done using the KoboCollect App on Android based devices linked to a cloud server. The results of school-level, participant-level, stool, and urine were recorded in four structured forms. The GPS coordinates of every PSU were noted. Data validation and backup procedures were monitored on a regular basis. Proper monitoring of the transmitted data was done during the study. Hardcopy backups were also maintained.

### 2.8 *Data Analyses*

Cleaning of data was done in Microsoft Excel. The prevalence between the categories of infection, i.e., based on sex, age groups, and LGAs were determined through descriptive statistics. WHO thresholds were used to classify endemicity; STH(< 2%, ≥ 2% to < 10%, ≥ 10% to < 20%, and ≥ 20%) and SCH (<10%, ≥ 10% to 49.9, and ≥ 50%) (WHO, 2024). The severity of the infections was determined based on WHO guidelines [7]. The 95% confidence intervals (CI) were used to calculate prevalence estimates. Chi-square (χ^2^) analysis was performed to test associations. Bivariate and multivariate logistic regression was used to estimate odds ratios (ORs) and adjusted odds ratios (aORs) with 95% CI. The significant level was established at p < 0.05. Statistical analysis was conducted using Statistical Package for Social Sciences (SPSS) version 27.

## 3 Results

### 3.1 Participant characteristics

A total of 6,431 SAC were examined (52.7% males; 47.3% females). The majority (62.2%) were aged 8–10 years, while 37.8% were aged 11–14 years (Table 1).

**Table 1:** Demographics of study participants.

Variables	N = 6431	%
Gender		
Male	3392	52.7
Female	3039	47.3
Age		
8-10	3998	62.2
11-14	2433	37.8

### 3.2 Prevalence of SCH and STH infection in 26 LGAs of the state

A total of 6431 students participated in this survey, comprising of 52.7% males (3392 individuals) and 47.3% females (3039 individuals), with 62.2% (3998 individuals) in the age group of 8–10 years and 37.8% (2433 individuals) in the age group of 11–14 years.

In this survey, samples from all SAC revealed they were not infected with *Schistosoma mansoni* and *Schistosoma haematobium*, 0.0% prevalence was recorded in all the PSUs across the 26 LGAs studied (Table 2). An overall prevalence of 27.62% [95% C.I. = 26.54 – 28.72%] (1776 students) was recorded among SAC for STH in this survey, with parasite-specific prevalence of 15.38% [95% C.I. = 14.52 – 16.28%] for hookworms, 15.10% [95% C.I. = 14.24 – 15.99%] for *Ascaris lumbricoides,* and 1.52% [95% C.I. = 1.25 – 1.85%] for *Trichuris trichiura*. Co-infection was highest between *Ascaris lumbricoides* and hookworm (3.39%), and least between *Trichuris trichiura* and hookworm (0.47%) (Table 2).

**Table 2:** Prevalence of *Schistosoma* spp. and STH by species.

Prevalence	NE	NI (%)	95% C.I.
*Schistosoma* spp	6431	0 (0.00)	NA
*Schistosoma mansoni*	6431	0 (0.00)	NA
*Schistosoma haematobium*	6431	0 (0.00)	NA
STH	6431	1776 (27.62)	26.54 – 28.72
*Ascaris lumbricoides*	6431	971 (15.10)	14.24 – 15.99
*Trichuris trichiura*	6431	98 (1.52)	1.25 – 1.85
Hookworm	6431	989 (15.38)	14.52 – 16.28
Co-infection			
*Ascaris spp* + *Trichuris spp*	6431	55 (0.86)	0.66 – 1.11
*Ascaris spp* + Hookworm	6431	218 (3.39)	2.97 – 3.86
*Trichuris spp* + Hookworm	6431	30 (0.47)	0.32 – 0.67
*Ascaris spp* + *Trichuris spp* + Hookworm	6431	21 (0.33)	0.21 – 0.50

NE – Number Examined, NI – Number Infected, NA – Not Applicable.

**Fig 2:**
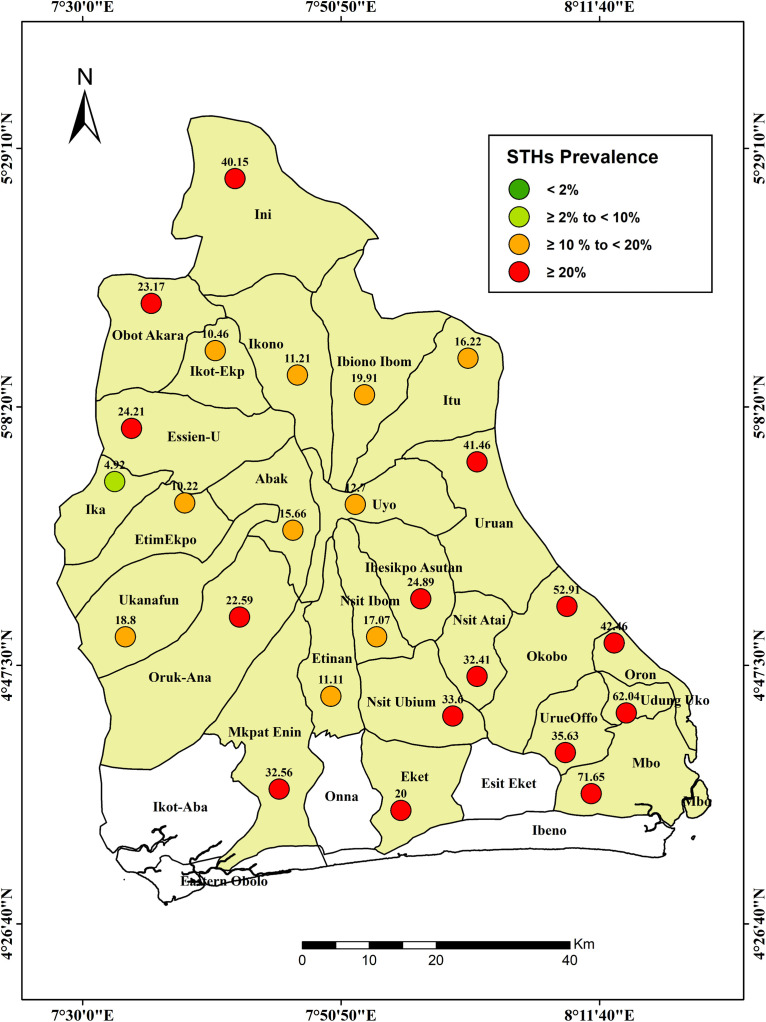
Prevalence of Soil-transmitted helminths across the 26 endemic LGAs of Akwa Ibom State, Nigeria. This figure was created by the authors on ArcMap 10.8 software. Available at https://desktop.arcgis.com/en/arcmap/index.html. The Nigeria and Akwa Ibom State shapefile was obtained from https://data.humdata.org/dataset/geoboundaries-admin-boundaries-for-nigeria, a publicly accessible database and are licensed under CC BY 4.0 (https://data.humdata.org). The authors allow unrestricted reuse of this map provided that proper credit is given to the source.

**Fig 3:**
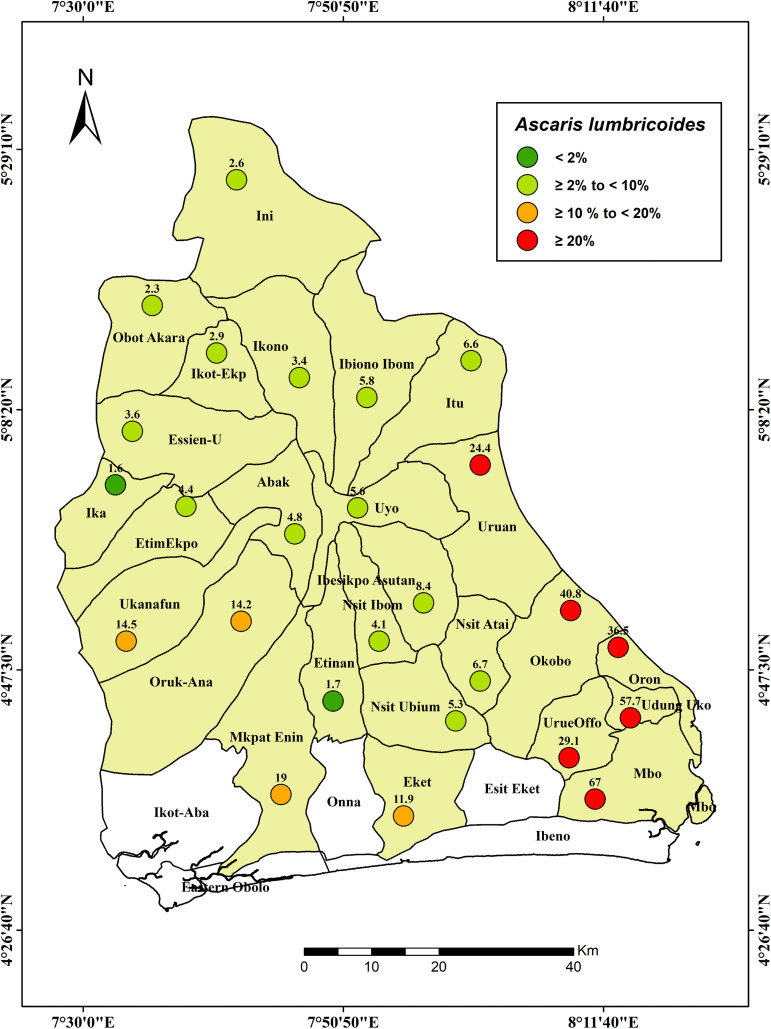
Prevalence of *Ascaris lumbricoides* across the 26 endemic LGAs of Akwa Ibom State, Nigeria. This figure was created by the authors on ArcMap 10.8 software. Available at https://desktop.arcgis.com/en/arcmap/index.html. The Nigeria and Akwa Ibom State shapefile was obtained from https://data.humdata.org/dataset/geoboundaries-admin-boundaries-for-nigeria, a publicly accessible database and are licensed under CC BY 4.0 (https://data.humdata.org). The authors allow unrestricted reuse of this map provided that proper credit is given to the source.

**Fig 4:**
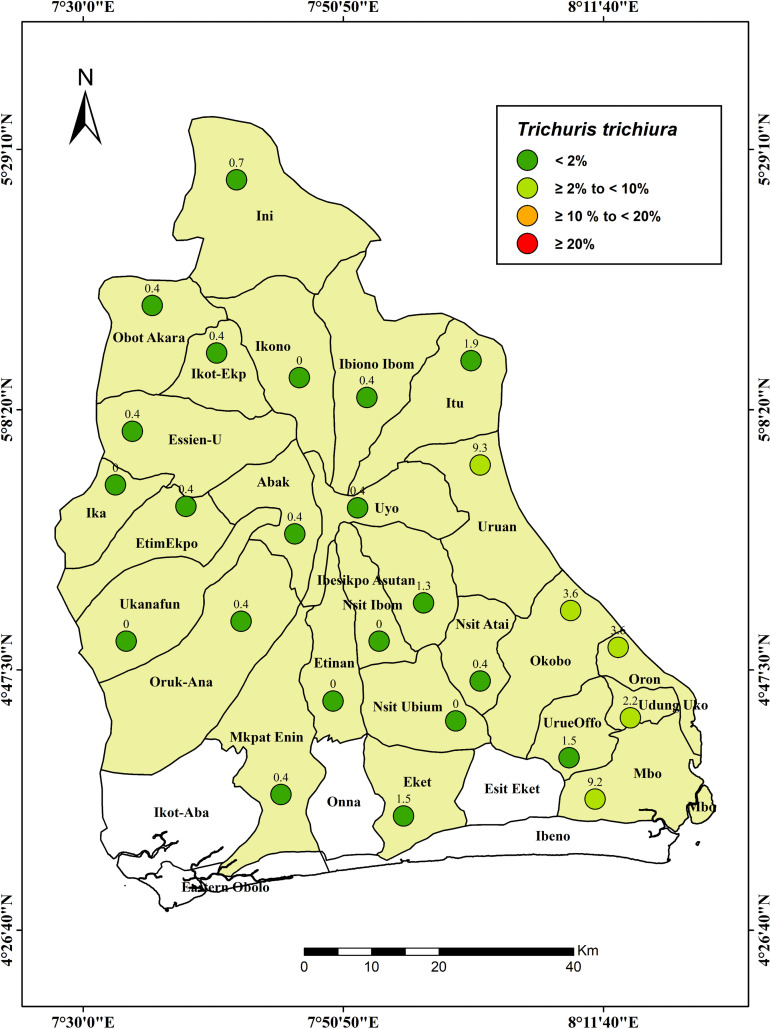
Prevalence of *Trichuris trichiura* across the 26 endemic LGAs of Akwa Ibom State, Nigeria. This figure was created by the authors on ArcMap 10.8 software. Available at https://desktop.arcgis.com/en/arcmap/index.html. The Nigeria and Akwa Ibom State shapefile was obtained from https://data.humdata.org/dataset/geoboundaries-admin-boundaries-for-nigeria, a publicly accessible database and are licensed under CC BY 4.0 (https://data.humdata.org). The authors allow unrestricted reuse of this map provided that proper credit is given to the source.

**Fig 5:**
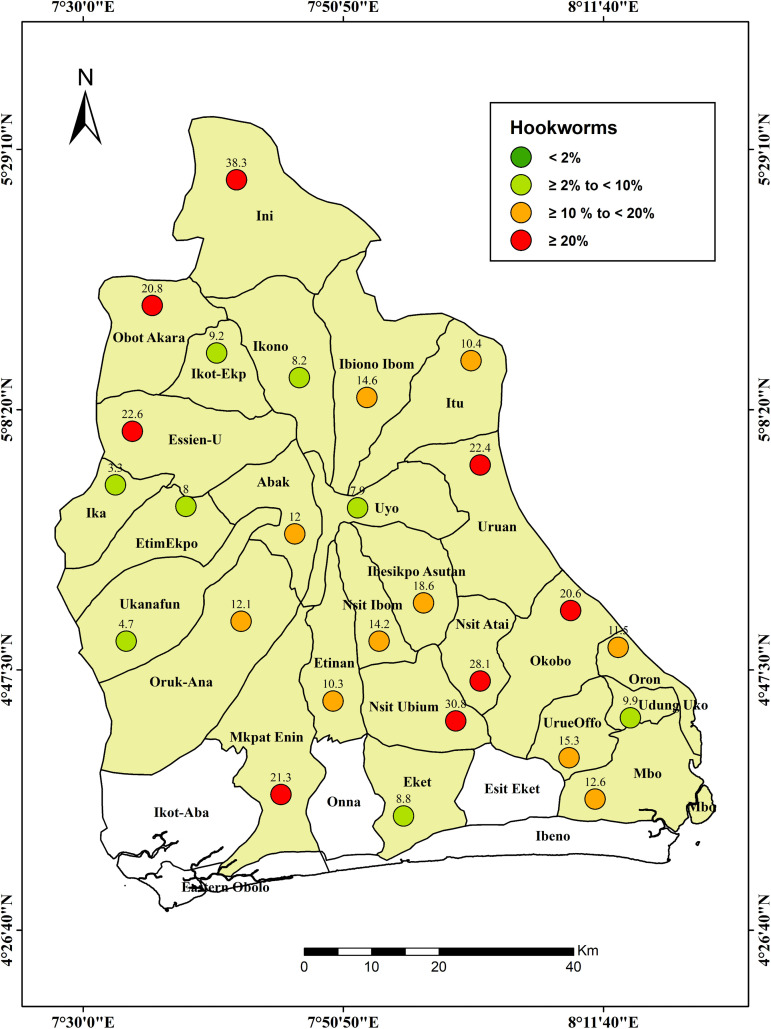
Prevalence of Hookworms across the 26 Endemic LGAs of Akwa Ibom State, Nigeria. This figure was created by the authors on ArcMap 10.8 software. Available at https://desktop.arcgis.com/en/arcmap/index.html. The Nigeria and Akwa Ibom State shapefile was obtained from https://data.humdata.org/dataset/geoboundaries-admin-boundaries-for-nigeria, a publicly accessible database and are licensed under CC BY 4.0 (https://data.humdata.org). The authors allow unrestricted reuse of this map provided that proper credit is given to the source.

The result of STH prevalence by LGA is presented in the Table 3. Mbo (71.65%) had the highest prevalence, followed by Udung Uko (62.04%), and the least prevalence was recorded at Ika LGA (4.92%). Comparison of the prevalence among the LGAs was statistically significant (p < 0.05%) (Fig 2, Table 3). *Ascaris lumbricoides* infection was highest at Mbo LGA (67.0%, 175 children), followed by Udung Uko LGA (57.7%, 158 children) and Okobo (40.8%, 91 children), while the lowest prevalence was at Ika LGA (1.6%, 4 children). Prevalence of *Ascaris lumbricoides* was statistically significant (p < 0.001) across the LGAs (Table 4, Fig 3). *Trichuris trichiura* prevalence was highest at Uruan LGA (9.3%, 23 children), followed by Mbo LGA (9.2%, 24 children). Prevalence was also statistically significant across the LGA (p < 0.001) (Table 4, Fig 4). Hookworms infection was highest at Ini LGA (38.3%, 103 children), followed by Nsit Ubium LGA (30.8%, 76 children), while the lowest prevalence was recorded at Ika LGA (3.3%, 8 children), the variation in prevalence was statistically significant across the LGAs (p < 0.001) (Table 4, Fig 5).

**Table 3:** Prevalence of *Schistosoma* spp. and STH by LGA.

LGA	NE	NI (%) with *Schistosoma* spp.	NI (%) with STH	95% C.I. of STH
Abak	249	0 (0.00)	39 (15.66)	11.56 - 20.56
Eket	260	0 (0.00)	52 (20.00)	15.49 - 25.18
Essien Udim	252	0 (0.00)	61 (24.21)	19.23 - 29.77
Etim Ekpo	225	0 (0.00)	23 (10.22)	6.77 - 14.69
Etinan	234	0 (0.00)	26 (11.11)	7.57 - 15.61
Ibesikpo Asutan	237	0 (0.00)	59 (24.89)	19.72 - 30.68
Ibiono Ibom	226	0 (0.00)	45 (19.91)	15.11 - 25.48
Ika	244	0 (0.00)	12 (4.92)	2.72 - 8.18
Ikono	232	0 (0.00)	26 (11.21)	7.63 - 15.74
Ikot Ekpene	239	0 (0.00)	25 (10.46)	7.06 - 14.81
Ini	269	0 (0.00)	108 (40.15)	34.42 - 46.09
Itu	259	0 (0.00)	42 (16.22)	12.11 - 21.07
Mbo	261	0 (0.00)	187 (71.65)	65.96 - 76.85
Mkpat Enin	258	0 (0.00)	84 (32.56)	27.06 - 38.45
Nsit Atai	253	0 (0.00)	82 (32.41)	26.87 - 38.35
Nsit Ibom	246	0 (0.00)	42 (17.07)	12.77 - 22.15
Nsit Ubium	247	0 (0.00)	83 (33.60)	27.93 - 39.66
Obot Akara	259	0 (0.00)	60 (23.17)	18.34 - 28.58
Okobo	223	0 (0.00)	118 (52.91)	46.36 - 59.39
Oron	252	0 (0.00)	107 (42.46)	36.47 - 48.62
Oruk Anam	239	0 (0.00)	54 (22.59)	17.64 - 28.21
Udung Uko	274	0 (0.00)	170 (62.04)	56.20 - 67.64
Ukanafun	234	0 (0.00)	44 (18.80)	14.20 - 24.18
Uruan	246	0 (0.00)	102 (41.46)	35.44 - 47.69
Urueoffong/Oruko	261	0 (0.00)	93 (35.63)	30.01 - 41.58
Uyo	252	0 (0.00)	32 (12.70)	9.02 - 17.23
Total	**6431**	**0 (0.00)**	**1776 (27.62)**	**26.53 - 28.72**
χ^2^			881.31	
df			25	
p Value			<0.001	

NE – Number Examined, NI – Number Infected.

**Table 4:** Prevalence of *Ascaris lumbricoides*, *Trichuris trichiura* and Hookworms by LGA.

LGA	NE	*Ascaris lumbricoides* (%)	*Trichuris trichiura* (%)	Hookworms (%)
Abak	249	12 (4.8)	1 (0.4)	30 (12.0)
Eket	260	31 (11.9)	4 (1.5)	23 (8.8)
Essien Udim	252	9 (3.6)	1 (0.4)	57 (22.6)
Etim Ekpo	225	10 (4.4)	1 (0.4)	18 (8.0)
Etinan	234	4 (1.7)	0 (0.0)	24 (10.3)
Ibesikpo Asutan	237	20 (8.4)	3 (1.3)	44 (18.6)
Ibiono Ibom	226	13 (5.8)	1 (0.4)	33 (14.6)
Ika	244	4 (1.6)	0 (0.0)	8 (3.3)
Ikono	232	8 (3.4)	0 (0.0)	19 (8.2)
Ikot Ekpene	239	7 (2.9)	1 (0.4)	22 (9.2)
Ini	269	7 (2.6)	2 (0.7)	103 (38.3)
Itu	259	17 (6.6)	5 (1.9)	27 (10.4)
Mbo	261	175 (67.0)	24 (9.2)	33 (12.6)
Mkpat Enin	258	49 (19.0)	1 (0.4)	55 (21.3)
Nsit Atai	253	17 (6.7)	1 (0.4)	71 (28.1)
Nsit Ibom	246	10 (4.1)	0 (0.0)	35 (14.2)
Nsit Ubium	247	13 (5.3)	0 (0.0)	76 (30.8)
Obot Akara	259	6 (2.3)	1 (0.4)	54 (20.8)
Okobo	223	91 (40.8)	8 (3.6)	46 (20.6)
Oron	252	92 (36.5)	9 (3.6)	29 (11.5)
Oruk Anam	239	34 (14.2)	1 (0.4)	29 (12.1)
Udung Uko	274	158 (57.7)	6 (2.2)	27 (9.9)
Ukanafun	234	34 (14.5)	0 (0.0)	11 (4.7)
Uruan	246	60 (24.4)	23 (9.3)	55 (22.4)
Urueoffong/Oruko	261	76 (29.1)	4 (1.5)	40 (15.3)
Uyo	252	14 (5.6)	1 (0.4)	20 (7.9)
**Total**	**6431**	**971 (15.1)**	**98 (1.5)**	**989 (15.4)**
χ^**2**^		1567.56	261.008	341.296
Df		25	25	26
p Value		<0.001*	<0.001*	<0.001*

NE – Number Examined.

Age specific prevalence revealed that school children in the age group of 11–14 years, 31.77% [95% C.I. = 29.95 – 33.64%] had higher prevalence than those in the age group of 8–10 years [95% C.I. = 23.77 – 26.46%] (Table 5). Comparison between the age groups was statistically significant (p < 0.05). Sex specific prevalence revealed a higher prevalence in males [29.30%, 95% C.I = 27.80 – 30.86%] than in females [25.73%, 95% C.I. = 24.24 – 27.35%], comparison of prevalence between sexes was statistically significant (p < 0.05) (Table 5).

**Table 5:** Overall prevalence of *Schistosoma* spp. and STH by age groups and sex.

Age and Sex	NE	NI (%) with *Schistosoma* spp.	NI (%) with STH	95% C.I. of STH
Age Groups (Years)				
8-10	3998	0 (0.00)	1003 (25.09)	23.77 - 26.46
11-14	2433	0 (0.00)	773 (31.77)	29.95 - 33.65
	**6431**	**0 (0.00)**	**1776 (27.62)**	**26.54 - 28.72**
χ^2^		NA	33.804	
df		NA	1	
P Value		Na	<0.001*	
				
Sex				
Male	3392	0 (0.00)	994 (29.30)	27.80 - 30.86
Female	3039	0 (0.00)	782 (25.73)	24.24 - 27.35
	**6431**	**0 (0.00)**	**1776 (27.62)**	**26.54 - 28.72**
χ^2^		NA	10.232	
df		NA	1	
P Value		NA	<0.001*	

NE – Number Examined, NI – Number Infected, NA – Not Applicable.

### 3.3 STH infection load estimation

A total of 98.66% (958 school children) and 1.44% (13 school children) had light and moderate infection of *A. lumbricoides* respectively, had light infection, while 1.44% (14 individuals) had moderate infection. Also, 99.09% (980 school children) and 0.91% (9 school children) had light and moderate infections of hookworms. All 98 school children infected with *T. trichiura* had light infection. High intensity infection was not recorded across the 26 LGAs (Table 6).

**Table 6:** Intensity of STH infection.

LGA	NE	NI	*Ascaris lumbricoides*				*Trichuris trichiura*				Hookworms			
			n	L (1–4999 epg)	M (5000-49.999 epg)	H (≥50,000 epg)	n	L (1–999 epg)	M (1000–9,999 epg)	H (≥10,000 epg)	n	L (1–1999 epg)	M (2000–3999 epg)	H (≥4000 epg)
Abak	249	39	12	10 (83.3)	2 (16.7)	–	1	1 (100.0)	–	–	30	30 (12.0)	–	–
Eket	260	52	31	31 (100.0)	–	–	4	4 (100.0)	–	–	23	23 (8.8)	–	–
Essien Udim	252	61	9	8 (88.9)	1 (11.1)	–	1	1 (100.0)	–	–	57	57 (22.6)	–	–
Etim Ekpo	225	23	10	10 (100.0)	–	–	1	1 (100.0)	–	–	18	18 (8.0)	–	–
Etinan	234	26	4	4 (100.0)	–	–	0	–	–	–	24	24 (10.3)	–	–
Ibesikpo Asutan	237	59	20	20 (100.0)	–	–	3	3 (100.0)	–	–	44	42 (95.5)	2 (4.5)	–
Ibiono Ibom	226	45	13	13 (100.0)	–	–	1	1 (100.0)	–	–	33	33 (14.6)	–	–
Ika	244	12	4	4 (100.0)	–	–	0	–	–	–	8	8 (3.3)	–	–
Ikono	232	26	8	8 (100.0)	–	–	0	–	–	–	19	19 (8.2)	–	–
Ikot Ekpene	239	25	7	7 (100.0)	–	–	1	1 (100.0)	–	–	22	21 (95.5)	1 (4.5)	–
Ini	269	108	7	7 (100.0)	–	–	2	2 (100.0)	–	–	103	103 (38.3)	–	–
Itu	259	42	17	17 (100.0)	–	–	5	5 (100.0)	–	–	27	27 (10.4)	–	–
Mbo	261	187	175	174 (99.4)	1 (0.57)	–	24	24 (100.0)	–	–	33	33 (12.6)	–	–
Mkpat Enin	258	84	49	48 (98.0)	1 (2.0)	–	1	1 (100.0)	–	–	55	55 (21.3)	–	–
Nsit Atai	253	82	17	17 (100.0)	–	–	1	1 (100.0)	–	–	71	71 (28.1)	–	–
Nsit Ibom	246	42	10	10 (100.0)	–	–	0	–	–	–	35	35 (14.2)	–	–
Nsit Ubium	247	83	13	13 (100.0)		–	0	–	–	–	76	76 (30.8)	–	–
Obot Akara	259	60	6	6 (100.0)	–	–	1	1 (100.0)	–	–	54	54 (20.8)	–	–
Okobo	223	118	91	91 (100.0)	–	–	8	8 (100.0)	–	–	46	46 (20.6)	–	–
Oron	252	107	92	91 (98.9)	1 (1.1)	–	9	9 (100.0)	–	–	29	29 (11.5)	–	–
Oruk Anam	239	54	34	34 (100.0)	–	–	1	1 (100.0)	–	–	29	29 (12.1)	–	–
Udung Uko	274	170	158	154 (97.5)	4 (2.5)	–	6	6 (100.0)	–	–	27	25 (92.6)	2 (7.4)	–
Ukanafun	234	44	34	34 (100	–	–	0	–	–	–	11	11 (4.7)	–	–
Uruan	246	102	60	57 (95.0)	3 (5.0)	–	23	23 (100.0)	–	–	55	52 (94.5)	3 (5.5)	–
Urueoffong/Oruko	261	93	76	76 (100.0)	–	–	4	4 (100.0)	–	–	40	40 (15.3)	–	–
Uyo	252	32	14	14 (100.0)	–	–	1	1 (100.0)	–	–	20	19 (95.0)	1 (5.0)	–
**Total**	**6431**	**1776**	**971**	**958 (98.66)**	**13 (1.34)**	**–**	**98**	**98 (100.0)**	**–**	**–**	**989**	**980 (99.09)**	**9 (0.91)**	**–**

NE – Number Examined, NI – Number Infected, n – Number infected with species of STHs.

### 3.4 Comparison between baseline and current prevalence of STH in Akwa Ibom State

**Fig 6:**
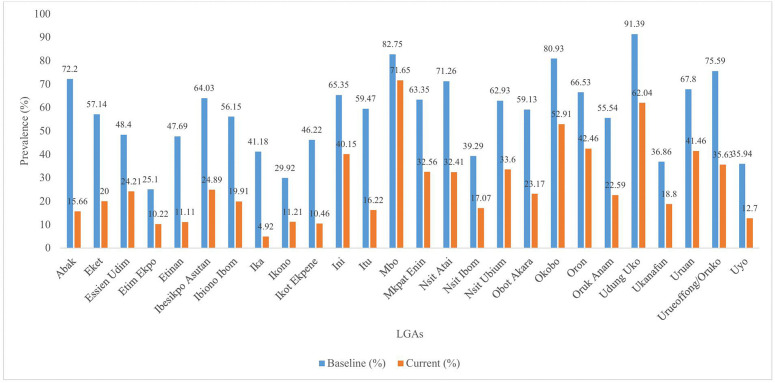
Comparison between baseline and current prevalence of soil transmitted helminths in 26 endemic LGAs of Akwa Ibom State, Nigeria.

An overall prevalence of 27.62% was recorded in the current survey of October 2025, with prevalence (ranges: 4.92% to 71.65%) compared to baseline prevalence of 57.77% (range: 25.1% to 91.39%) for Akwa Ibom State, with a 30.15% reduction in prevalence. Across the 26 LGAs surveyed, there was a reduction in prevalence compared to the baseline. Although some LGAs recorded a significant reduction than others, Abak LGA reported the highest significant reduction in prevalence with over 56.54% reduction (15.66% current, 72.2% baseline), followed by Itu LGA with 43.25% reduction (16.22% current, 59.47% baseline), while Mbo LGA has the least reduction in prevalence with 11.1% reduction (71.65% current, 82.75% baseline) (Fig 6, Table 7).

**Table 7:** Comparison between baseline and current prevalence of STH in Akwa Ibom State.

LGAs	Baseline (%)	Current (%)	Difference (%) = Baseline - Current
Abak	72.2	15.66	56.54
Eket	57.14	20	37.14
Essien Udim	48.4	24.21	24.19
Etim Ekpo	25.1	10.22	14.88
Etinan	47.69	11.11	36.58
Ibesikpo Asutan	64.03	24.89	39.14
Ibiono Ibom	56.15	19.91	36.24
Ika	41.18	4.92	36.26
Ikono	29.92	11.21	18.71
Ikot Ekpene	46.22	10.46	35.76
Ini	65.35	40.15	25.2
Itu	59.47	16.22	43.25
Mbo	82.75	71.65	11.1
Mkpat Enin	63.35	32.56	30.79
Nsit Atai	71.26	32.41	38.85
Nsit Ibom	39.29	17.07	22.22
Nsit Ubium	62.93	33.6	29.33
Obot Akara	59.13	23.17	35.96
Okobo	80.93	52.91	28.02
Oron	66.53	42.46	24.07
Oruk Anam	55.54	22.59	32.95
Udung Uko	91.39	62.04	29.35
Ukanafun	36.86	18.8	18.06
Uruan	67.8	41.46	26.34
Urueoffong/Oruko	75.59	35.63	39.96
Uyo	35.94	12.7	23.24
**Total**	**57.77**	**27.62**	**30.15**
Range	25.1 to 91.39	4.92 to 71.65	

### 3.5 Risk factors of STH and infection status

The study also examined the socio-demographic characteristics and WASH factors in relation to the infection status. The risk of acquiring STH infections was significantly associated with sex, age group of school children, type of toilet facility, when and where children urinate and defecate in schools (Table 8). Male school children had a higher likelihood of being infected with STH (aOR = 1.196, 95% CI: 1.072 – 1.335). Children in the age group of 11–14 years (aOR = 1.391, 95% C.I.: 1.244 – 1.554) are at higher risk of being infected with STH. School children that uses dry pit (aOR = 1.464, 95% C.I. = 1.239 – 1.731), urinate around school (aOR = 2.620, 95% C.I.: 2.362 – 2.929), defecate around school (aOR = 1.238, 95% C.I. = 1.097 – 1.396) or wait to defecate after school (aOR = 1.830, 95% C.I. = 1.365 – 2.454) had higher likelihood of being infected with STH. Also, school children that drink water from borehole with hand pump (aOR = 1.666, 95% C.I. = 1.412 – 1.965) and those that drink from surface water (aOR = 1.731, 95% C.I. = 1.486 – 2.017) had higher likelihood of being infected with STH (Table 8).

**Table 8:** Bivariate analysis of risk factors associated with STH infection in the study area.

Characteristics	NE	NI (%)	χ^2^	*p*	OR (95% C.I.)	AOR (95% C.I.)
Sex						
Male	3392	994 (29.30)	10.232	0.001*	1.196 (1.072 – 1.335)*	1.196 (1.072 – 1.335)*
Female	3039	782 (25.73)			1	0.836 (0.789 – 0.933)
Age (years)						
8 – 10	3998	1003 (25.09)	33.804	< 0.001*	1	0.719 (0.643 – 0.804)
11 - 14	2433	773 (31.77)			1.391 (1.244 – 1.554)*	1.391 (1.244 – 1.554)*
Hand washing after defecation						
No	1040	294 (28.27)	0.265	0.607ns	1.040 (0.897 – 1.205)	1.040 (0.897 – 1.205)
Yes	5391	1482 (27.49)			1	0.962 (0.830 – 1.115)
Use of soap and water to wash hands						
No	1790	388 (21.68)	2.620	0.106ns	0.897 (0.787 – 1.023)	0.897 (0.787 – 1.023)
Yes	4641	1094 (23.57)			1	1.115 (0.977 – 1.271)
Toilet facility used by household						
Bush	163	43 (26.38)	25.644	< 0.001*	1.706 (1.155 – 2.520)*	0.938 (0.659 – 1.334)
Bucket	18	4 (22.22)			1.361 (0.442 – 4.190)	0.748 (0.246 – 2.277)
Composting	5	0 (0.00)			NA	NA
Container	2	1 (50.00)			4.762 (0.296 – 76.553)	2.622 (0.164 – 41.944)
Dry pit	5506	1577 (28.64)			1.911 (1.589 – 2.299)*	1.464 (1.239 – 1.731)*
Flush/WC	734	151 (41.14)			1	0.649 (0.538 – 0.783)
Hanging	3	0 (0.00)			NA	NA
When you are at school, where do you usually go to urinate?						
Around the school compound	2561	786 (30.69)	62.977	< 0.001*	1.213 (1.084 – 1.358)*	2.620 (2.362 – 2.929)*
I wait/hold	40	16 (40.00)			1.827 (0.966 – 3.454)	1.754 (0.930 – 3.310)
In a nearby home or bush	282	27 (9.58)			0.290 (0.194 – 0.434)	0.266 (0.179 – 0.398)
In the school toilet	3508	938 (26.74)			1	0.908 (0.814 – 1.013)
Outside the school compound	40	9 (22.50)			0.795 (0.377 – 1.677)	0.760 (0.361 – 1.599)
When you are at school, where do you usually go to defecate?						
Around the school compound	1758	541 (30.77)	49.754	< 0.001*	1.225 (1.080 – 1.388)*	1.238 (1.097 – 1.396)*
I wait/hold	192	78 (40.63)			1.885 (1.400 – 2.537)*	1.830 (1.365 – 2.454)*
In a nearby home or bush	615	118 (19.19)			0.653 (0.527 – 0.808)	0.595 (0.483 – 0.734)
In the school toilet	3612	962 (26.63)			1	0.894 (0.801 – 0.998)
Outside the school compound	254	77 (30.32)			1.198 (0.908 – 1.582)	1.147 (0.872 – 1.507)
What is the main source of drinking water for members of your household?						
Hand Pump	689	258 (37.45)	135.009	<0.001*	1	1.666 (1.412 – 1.965)*
Piped into private dwelling	119	23 (19.33)			0.400 (0.248 – 0.647)	0.623 (0.394 – 0.985)
Piped into private yard	211	42 (19.91)			0.415 (0.286 – 0.602)	0.643 (0.457 – 0.906)
Protected dug well	2033	558 (27.45)			0.632 (0.527 – 0.759)	0.988 (0.878 – 1.111)
Protected spring	680	157 (23.09)			0.502 (0.396 – 0.635)	0.766 (0.635 – 0.924)
Public-piped water	1075	211 (19.63)			0.408 (0.329 – 0.506)	0.592 (0.503 – 0.695)
Rainwater	338	98 (29.00)			0.682 (0.515 – 0.904)	1.074 (0.844 – 1.368)
Sachet	11	4 (36.36)			0.955 (0.277 – 3.293)	1.499 (0.438 – 5.127)
Surface water	825	313 (37.94)			1.021 (0.829 – 1.258)	1.731 (1.486 – 2.017)*
Tanker truck	2	1 (50.00)			1.671 (0.104 – 26.824)	2.622 (0.164 – 41.944)
Unprotected dug well	16	7 (43.75)			1.299 (0.478 – 3.531)	2.043 (0.760 – 5.493)
Unprotected spring	432	104 (24.07)			0.530 (0.405 – 0.693)	0.821 (0.654 – 1.030)
Taken deworming tablet before?						
No	240	52 (21.70)	4.415	0.036*	0.717 (0.525 – 0.979)	0.717 (0.525 – 0.979)
Yes	6191	1724 (27.80)			1	1.395 (1.021 – 1.906)*
Wearing any footwear (sandals, shoes, slippers e.t.c.)						
No	64	11 (17.19)	3.517	0.061ns	0.541 (0.282 – 1.038)	0.541 (0.282 – 1.038)
Yes	6367	1765 (27.72)			1	1.848 (0.963 – 3.546)

ns – Not significant at p > 0.05, * - Significant at p ≤ 0.05, NA – Not applicable.

### 3.6 Programmatic interpretation of results

**Fig 7:**
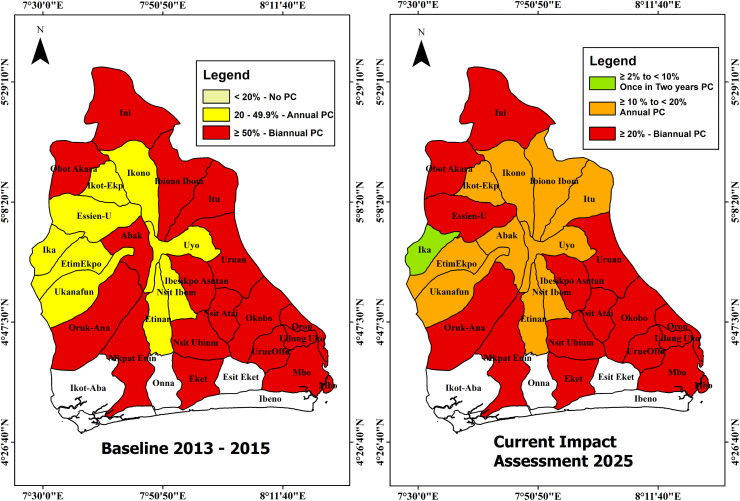
Endemicity map of baseline and current impact assessment in 26 LGAs of Akwa Ibom State. This figure was created by the authors on ArcMap 10.8 software. Available at https://desktop.arcgis.com/en/arcmap/index.html. The Nigeria and Akwa Ibom State shapefile was obtained from https://data.humdata.org/dataset/geoboundaries-admin-boundaries-for-nigeria, a publicly accessible database and are licensed under CC BY 4.0 (https://data.humdata.org). The authors allow unrestricted reuse of this map provided that proper credit is given to the source.

The results of this impact assessment has shown significant decrease in the STH prevalence thresholds across the 26 LGAs of Akwa Ibom State. Out of 17 LGAs in the threshold of ≥50% at baseline, 14 remained in the threshold of ≥20% while 3 LGAs moved down to the threshold of ≥10 to <20%. Also, 9 LGAs in the threshold of 20 to 49.9% at baseline, 1 LGA moved down to the threshold of ≥2 to <10%, 7 LGAs moved down to the threshold of ≥10 to <20% while 1 LGA remained in the threshold of ≥20%. Based on this report, 1 LGA requires MDA once in 2 years for a period of 5 years with sustained surveillance activities, 10 LGAs requires MDA once yearly for a period of 5 years with sustained surveillance while 15 LGAs requires MDA twice yearly for a period of 5 years with sustained surveillance activities (Table 9, Fig 7).

**Table 9:** Programmatic interpretation of impact assessment across the 26 LGAs.

Prevalence Threshold (2025)	Baseline Prevalence (2013–2015)			WHO Recommendations/PC Requirement on Current Impact Assessment
	0 to 19.9%	20 to 49.9%	≥ 50%	
< 2%	None	None	None	PC Not Required. Surveillance should be sustained
≥ 2% to < 10%	None	Ika	None	1 LGA: MDA should be conducted once in 2 years for a period of 5 years, with surveillance activities sustained.
≥ 10% to < 20%	None	Etim Ekpo, Etinan, Ikono, Ikot Ekpene, Nsit Ibom, Ukanafun, Uyo	Abak, Ibiono Ibom, Itu	10 LGAs: MDA should be carried out once yearly for a period of 5 years, with surveillance activities sustained.
≥ 20%	None	Essien Udim	Eket, Ibesikpo Asutan, Ini, Mbo, Mkpat Enin, Nsit Atai, Nsit Ubium, Obot Akara, Okobo, Oron, Oruk Anam, Udung Uko, Uruan, Urueoffong/Oruko	15 LGAs: MDA should be carried out twice yearly for a duration of 5 years, with surveillance activities sustained.

## 4 Discussion

The WHO targeted the elimination of schistosomiasis and soil-transmitted helminthiasis by 2030. In view of this, states that have conducted several rounds of chemotherapy need to assess the status of these diseases through conducting impact assessments to review the control strategies. In 2024, impact assessment was conducted in 5 LGAs co-endemic for STH and SCH following a minimum of five effective rounds of mass drug administration.

The current impact assessment was carried in October 2025, to understand the current distribution of schistosomiasis and soil-transmitted helminthiasis across 26 LGAs. The observation from the current survey carried out in 26 LGAs of Akwa Ibom State revealed 0.0% prevalence of *Schistosoma* spp. and 27.62% overall prevalence of STH, indicating a 30.15% significant reduction in the overall prevalence of STH infection compared to the baseline survey of 2014. The state achieved effective programmatic action against schistosomiasis across the 26 LGAs, with all the wards visited having a prevalence threshold below the WHO recommendation. The state also recorded significant achievement in the reduction of prevalence of STH with a 30% reduction in overall prevalence, and varying reduction in prevalence across the LGAs with annual PC required in 1 LGA (Ika) once in 2 years, annual PC required in 10 LGAs (Abak, Etim Ekpo, Etinan, Ibiono Ibom, Ikono, Ikot Ekpene, Itu, Nsit Ibom, Ukanafun, and Uyo) for 5 years and biannual PC required in 15 LGAs (Essien Udim, Eket, Ibesikpo Asutan, Ini, Mbo, Mkpat Enin, Nsit Atai, Nsit Ubium, Obot Akara, Okobo, Oron, Oruk Anam, Udung Uko, Uruan, Urueoffong/Oruko) for 5 years. However, for STH, no LGA has met the WHO treatment threshold of less than 2% for stopping MDA, which mandates the need to intensify effort in the control of STH [15]. Despite the significant reduction in the prevalence of STH, factors such as lack of drugs, delayed supply chain, inability to conduct 2 rounds of MDA in some LGAs requiring such regimen must have affected the high prevalence observed in some LGAs.

The persistent dominance of hookworms (15.38%) is a significant public health concern, particularly due to its direct association with anaemia and cognitive impairment in children. In Ondo State, Nigeria [16], a study reported *Trichuris trichiura* as the most dominant species of STHs. The difference might be due to factors such as ecological and environmental differences. The observed statistically significant difference in infection rates between males and females suggests differential exposure to environmental risk factors, which must inform targeted behavioural change communication strategies. A similar observation was reported in a previous study in Plateau State [17]. Children in the age group of 11–14 years were affected more, indicating exposure to more high-risk environments and behaviours associated with that age. A study reported the same pattern for that age group of 11–14 years in Ondo State, Nigeria [1].

The survey confirms heterogeneity in prevalence with significant variation in the distribution of the parasites across the LGAs, which indicates the need to identify key elements resulting to the spatial variation in prevalence across the state, a programmatic challenge that should be tailored alongside chemotherapy for an intensified and concerted intervention package. Such packages include identifying non-functional basic water, sanitation and hygiene infrastructures, improvement in access and use of WASH through behavioural change, health and nutrition education, among other. The WHO recent guidelines emphasize on the need for improved access to water, sanitation and hygiene infrastructures [7].

Availability of toilets in schools, and the type of toilet available in the school environment for defecation, was a significant risk factor influencing the prevalence of STH in Akwa Ibom State. Similar observation was reported in Ekiti State [18], Kogi State [19] and Cross River State [20]. Also, clean-up behaviours in schools were found to be a significant contributor of infection. Children who urinated or defecated freely around the school compound had the highest rates of infection. This observation implies that a lack of school sanitation facilities, poor maintenance, or overcrowding could be making a significant contribution to the transmission. Enhancement of school WASH and better behaviour change communication among the pupils are still important actions that should be incorporated into the control programs [21]. Failure and ineffective performance of the control program caused by lack of access to WASH have been reported in different countries [22–25].

In general, this assessment confirms that the transmission of soil-transmitted helminth parasites in these communities is influenced by the combination of environmental, behavioural, and infrastructural-related elements. The responses to the determinants must be integrated in health, education, water, hygiene and sanitation. The integration and utilization of the novel diagnostic technologies, such as the AiDx digital microscope, has proven to be a good complement to microscopy in accelerating laboratory analysis time and enhancing data throughput for time-sensitive programmatic decisions [13,14,18].

## 5 Conclusion

Preventive chemotherapy (PC) program for schistosomiasis and soil-transmitted helminthiasis (STH) in Akwa Ibom state has proven highly effective for schistosomiasis with 0.0% prevalence compared to baseline of 0.0 to 1.2% but partly effective for soil-transmitted helminthiasis with overall prevalence of 27.62% (ranges from 4.92 – 71.65%) compared to baseline of 25.1 to 91.4% with a 30.15% reduction in prevalence. The survey documented a reduction in STH prevalence across all 26 LGAs; 1 LGA require PC once in two years, 10 LGAs require annual PC, and 15 LGAs require biannual PC. For SCH, continued case management is recommended across all 26 LGAs. The widespread absence of functional WASH facilities in schools remains a critical risk factor threatening the long-term sustainability of MDA-driven gains. These findings underscore the imperative for sustained treatment and control programmes, underpinned by substantive improvements in water, sanitation, and hygiene infrastructure.

## Supporting information

S1 Data Akwa Ibom State 2025 Survey on schistosomiasis amd soil transmitted helminths. This dataset contains laboratory results and risk factors associated with Schistosomiasis and Soil-transmitted helminths in 26 Local Government Areas of Akwa Ibom State.
(XLSX)
