# Supplementary material for: Impact assessment of effective rounds of mass drug administration against schistosomiasis and soil transmitted helminthiasis among school age children in Akwa Ibom State, Nigeria
Source: PLoS Negl Trop Dis. 2026 Aug 7;20(8):e0014603. doi: 10.1371/journal.pntd.0014603 (PMC13450779; doi:10.1371/journal.pntd.0014603)
Supplement: Supplementary file 1 [file pntd.0014603.s002.pdf]

PLOS Neglected Tropical Diseases

Impact Assessment of Effective Rounds of Mass Drug Administration against Schistosomiasis and Soil Transmitted Helminthiasis among School Age Children in Akwa Ibom State, Nigeria

--Manuscript Draft--

**Manuscript Number:**	PNTD-D-26-00512
**Full Title:**	Impact Assessment of Effective Rounds of Mass Drug Administration against Schistosomiasis and Soil Transmitted Helminthiasis among School Age Children in Akwa Ibom State, Nigeria
**Short Title:**	Impact Assessment of Schistosomiasis and Soil-Transmitted Helminthiasis in Akwa Ibom State
**Article Type:**	Research Article
**Keywords:**	Schistosomiasis; Soil-transmitted helminthiasis; Akwa Ibom; Nigeria
**Abstract:**	

Background

Schistosomiasis (SCH) and Soil-transmitted helminthiasis (STH) remain major neglected tropical diseases across sub-Saharan Africa. In Akwa Ibom State, preventive chemotherapy (PC) has been implemented in endemic Local Government Areas (LGAs). This study assessed the impact of five effective rounds of PC on the prevalence and intensity of SCH and STH in 26 endemic LGAs.

Methods

This study was carried out in 26 local government areas (LGAs) of Akwa Ibom State between September and October 2025. Prior to commencement, ethical approval was obtained from the research ethics committee. A school-based cross-sectional survey was employed. Schools were purposefully selected from endemic wards, and a total of 200 to 300 samples were collected per LGA from school children of both sexes aged 8-14 years. Samples were collected using a systematic random sampling method. Questionnaires were administered using electronic data forms. Stool samples collected were analyzed using the Kato-Katz technique, while urine samples were analyzed using the urine filtration method. Conventional microscopy alongside a novel AiDx digital microscope was used to examine the presence of any ova in the prepared specimen, enhancing diagnostic throughput and quality assurance. Data obtained were analyzed using descriptive statistics, chi-square test, bivariate logistic regression on SPSS, and maps were generated using ArcMap.

Results

No cases of Schistosoma mansoni or Schistosoma haematobium were detected across the 26 LGAs (0.0% prevalence). The overall prevalence of STH was 27.62% (95% CI: 26.54–28.72), ranging from 4.92% to 71.65% across LGAs. Species-specific prevalence was 15.38% (95% CI: 14.52–16.28) for hookworm, 15.10% (95% CI: 14.24–15.99) for Ascaris lumbricoides, and 1.52% (95% CI: 1.25–1.85) for Trichuris trichiura. Mbo (71.65%) and Udung Uko (62.04%) recorded the highest prevalences, whereas Ika (4.92%) had the lowest. Prevalence was significantly higher among children aged 11–14 years (31.77%; 95% CI: 29.95–33.64) compared with those aged 8–10 years (95% CI: 23.77–26.46), and among males (29.30%; 95% CI: 27.80–30.86) compared with females (25.73%; 95% CI: 24.24–27.35) ($p < 0.05$). Most infections were of light intensity (98.87%), with 1.13% moderate intensity. Compared with baseline data, STH prevalence declined by 30.15%. Significant risk factors included age (OR = 1.391; 95% CI: 1.244–1.554), sex (OR = 1.196; 95% CI: 1.072–1.335), type of toilet facility (OR = 1.706; 95% CI: 1.155–2.520), availability of school sanitation, and defecation practices (OR = 1.225; 95% CI: 1.080–1.388).

Conclusion

Schistosomiasis was not reported in 26 LGAs of Akwa Ibom. Soil-transmitted helminths

Powered by Editorial Manager® and ProduXion Manager® from Aries Systems Corporation

	had a significant reduction in prevalence, but with varying prevalence across the states. There is a need for sustained preventive chemotherapy against STH, especially in the south-eastern part of the State. Also, improvement in water, sanitation, and hygiene (WASH) infrastructures to create behavioural change, and strengthen the control efforts.
Additional Information:	
**Question**	**Response**
**Government Employee**<br><br>Are you or any of the contributing authors an employee of the United States government?<br><br>Manuscripts authored by one or more US Government employees are not copyrighted, but are licensed under a CC0 Public Domain Dedication, which allows unlimited distribution and reuse of the article for any lawful purpose. This is a legal requirement for US Government employees.<br><br>This will be typeset if the manuscript is accepted for publication.	No - No authors are employees of the U.S. government.
**Financial Disclosure**<br><br>Enter a financial disclosure statement that describes the sources of funding for the work included in this submission. Review the submission guidelines for detailed requirements. View published research articles from *PLOS Neglected Tropical Diseases* for specific examples.<br><br>This statement is required for submission and **will appear in the published article** if the submission is accepted. Please make sure it is accurate.	The author(s) received no specific funding for this work.

**Funded studies**<br>Enter a statement with the following details:<br>• Initials of the authors who received each award<br>• Grant numbers awarded to each author<br>• The full name of each funder<br>• URL of each funder website<br>• Did the sponsors or funders play any role in the study design, data collection and analysis, decision to publish, or preparation of the manuscript?<br><br>Did you receive funding for this work?	
**Competing Interests**<br><br>On behalf of all authors, disclose any competing interests that could be perceived to bias this work.<br><br>This statement will be typeset if the manuscript is accepted for publication.<br><br>Review the instructions link below and PLOS NTDs' competing interests policy to determine what information must be disclosed at submission.	The authors listed below declared that no conflict of interest exists.
**Data Availability**<br><br>Provide a **Data Availability Statement** in the box below. This statement should detail where the data used in this submission can be accessed. This statement will be typeset if the manuscript is accepted for publication.<br><br>Before publication, authors are required to make all data underlying their findings fully available, without restriction. Review our PLOS Data Policy page for detailed information on this policy. Instructions for writing your Data Availability statement can be accessed via the Instructions link below.	The data sets in this study are available from the corresponding author on reasonable request.

Click here to access/download;Manuscript;Manuscript.docx

Article Type: Original Research

Title: Impact Assessment of Effective Rounds of Mass Drug Administration against Schistosomiasis and Soil Transmitted Helminthiasis among School Age Children in Akwa Ibom State, Nigeria

Authors:

*Kenneth Nnamdi Opara[1], *Clement Ameh Yaro[1], Nnamso Williams Ikpeida[1], *Aliyu Mohammed[2], Hadiza Joy Marcu[2], Kingsley Azo[2], Salisu Ahmad[2], Esther Sunday Okon[2], Ellie Leaning[3], Moses Aderogba[3], Louise-Makau Barasa[3], Antia Etop[4], Imeh Udo Sylvester[4], Fatai Oyediran[5], Imabong O. Umah[5], Solomon M. Jacob[5], Gideon U. Ntuen[5], Veronica S. Augustine[5] and Halima Toro[5]

Authors Names and Full Addresses:

Kenneth Nnamdi Opara

[1]Department of Animal and Environmental Biology, Faculty of Biological Sciences, University of Uyo, Uyo, Akwa Ibom State, Nigeria. nkopara@yahoo.com, https://orcid.org/0000-0002-7422-0589

Clement Ameh Yaro

[1]Department of Animal and Environmental Biology, Faculty of Biological Sciences, University of Uyo, Uyo, Akwa Ibom State, Nigeria. yaro.ca@uniuyo.edu.ng, https://orcid.org/0000-0002-9214-6095

Nnamso Williams Ikpeida

[1]Department of Animal and Environmental Biology, Faculty of Biological Sciences, University of Uyo, Uyo, Akwa Ibom State, Nigeria. nnamsoikpeida.msc@uniuyo.edu.ng

Aliyu Mohammed

[2]Helen Keller International, Abuja, Nigeria. AMohammed@hki.org,

Hadiza Joy Marcu

[2]Helen Keller International, Abuja, Nigeria. HMarcus@hki.org,

Kingsley Azo
[2]Helen Keller International, Abuja, Nigeria. KAzu@hki.org

Salisu Ahmad
[2]Helen Keller International, Abuja, Nigeria. SAhmad@hki.org

Esther Sunday Okon
[2]Helen Keller International, Abuja, Nigeria. EOkon@hki.org

Ellie Leaning
[3]The END Fund. eleaning@end.org,

Moses Aderogba
[3]The END Fund. maderogba@end.org

Louise-Makau Barasa
[3]The END Fund. lmakau-barasa@endfund.org

Antia Etop
[4]Akwa Ibom State Ministry of Health, Uyo, Akwa Ibom State, Nigeria. etopantia50@gmail.com

Imeh Udo Sylvester
[4]Akwa Ibom State Ministry of Health, Uyo, Akwa Ibom State, Nigeria. drslyudoh@yahoo.com

Fatai Oyediran
[5]Federal Ministry of Health, Abuja. fatai_oyediran@yahoo.com

Imabong U. Umah
[5]Federal Ministry of Health, Abuja. imaumah@yahoo.com

Solomon M. Jacob
[5]Federal Ministry of Health, Abuja. owukpa2000@yahoo.com

Gideon U. Ntuen
[5]Federal Ministry of Health, Abuja. uduakgn@yahoo.com

Veronica S. Augustine
[5]Federal Ministry of Health, Abuja. Princessveemark@yahoo.com

Halima Toro
[5]Federal Ministry of Health, Abuja. Halima4toro@gmail.com

Corresponding Authors:
***Kenneth Nnamdi Opara**
[1]Department of Animal and Environmental Biology, Faculty of Biological Sciences, University of Uyo, Uyo, Akwa Ibom State, Nigeria. nkopara@yahoo.com, https://orcid.org/0000-0002-7422-0589

***Clement Ameh Yaro**
[1]Department of Animal and Environmental Biology, Faculty of Biological Sciences, University of Uyo, Uyo, Akwa Ibom State, Nigeria. yaro.ca@uniuyo.edu.ng, https://orcid.org/0000-0002-9214-6095

***Aliyu Mohammed**
[2]Helen Keller International, Abuja, Nigeria. AMohammed@hki.org

Abstract

Background: Schistosomiasis (SCH) and Soil-transmitted helminthiasis (STH) remain major neglected tropical diseases across sub-Saharan Africa. In Akwa Ibom State, preventive chemotherapy (PC) has been implemented in endemic Local Government Areas (LGAs). This study assessed the impact of five effective rounds of PC on the prevalence and intensity of SCH and STH in 26 endemic LGAs.

Methods: This study was carried out in 26 local government areas (LGAs) of Akwa Ibom State between September and October 2025. Prior to commencement, ethical approval was obtained from the research ethics committee. A school-based cross-sectional survey was employed. Schools were purposefully selected from endemic wards, and a total of 200 to 300 samples were collected per LGA from school children of both sexes aged 8-14 years. Samples were collected using a systematic random sampling method. Questionnaires were administered using electronic data forms. Stool samples collected were analyzed using the Kato-Katz technique, while urine samples were analyzed using the urine filtration method. Conventional microscopy alongside a novel AiDx digital microscope was used to examine the presence of any ova in the prepared specimen, enhancing diagnostic throughput and quality assurance. Data obtained were analyzed using descriptive statistics, chi-square test, bivariate logistic regression on SPSS, and maps were generated using ArcMap.

Results: No cases of *Schistosoma mansoni* or *Schistosoma haematobium* were detected across the 26 LGAs (0.0% prevalence). The overall prevalence of STH was 27.62% (95% CI: 26.54–28.72), ranging from 4.92% to 71.65% across LGAs. Species-specific prevalence was 15.38% (95% CI: 14.52–16.28) for hookworm, 15.10% (95% CI: 14.24–15.99) for *Ascaris lumbricoides*, and 1.52% (95% CI: 1.25–1.85) for *Trichuris trichiura*. Mbo (71.65%) and Udung Uko (62.04%) recorded the highest prevalences, whereas Ika (4.92%) had the lowest. Prevalence was significantly higher among children aged 11–14 years (31.77%; 95% CI: 29.95–33.64) compared with those aged 8–10 years (95% CI: 23.77–26.46), and among males (29.30%; 95% CI: 27.80–30.86) compared with females (25.73%; 95% CI: 24.24–27.35) ($p < 0.05$). Most infections were of light intensity (98.87%), with 1.13% moderate intensity. Compared with baseline data, STH prevalence declined by 30.15%. Significant risk factors included age (OR = 1.391; 95% CI: 1.244–1.554), sex (OR = 1.196; 95% CI: 1.072–1.335), type of toilet facility (OR = 1.706; 95% CI: 1.155–2.520), availability of school sanitation, and defecation practices (OR = 1.225; 95% CI: 1.080–1.388).

Conclusion: Schistosomiasis was not reported in 26 LGAs of Akwa Ibom. Soil-transmitted helminths had a significant reduction in prevalence, but with varying prevalence across the

states. There is a need for sustained preventive chemotherapy against STH, especially in the south-eastern part of the State. Also, improvement in water, sanitation, and hygiene (WASH) infrastructures to create behavioural change, and strengthen the control efforts.

Keywords: Schistosomiasis, Soil-transmitted helminthiasis, Akwa Ibom, Nigeria

Author Summary

This study assessed the impact of 5 rounds of mass drug administration (MDA) on schistosomiasis and soil-transmitted helminthiasis (STH) among 6,431 school-aged children. The results show schistosomiasis has been eliminated, with no detected cases. STH prevalence dropped by 30.15% to 27.62%, but disparities persist across regions (4.92% to 71.65%). Hookworm and *Ascaris lumbricoides* are the main STH species. Risk factors for STH include age 11 to 14 years, male, poor sanitation and inadequate toilet facilities. The MDA effort succeeded in eliminating schistosomiasis, but sustained interventions are required for STH, along with improved WASH infrastructures and behavioural changes to meet the WHO 2030 targets.

1. Introduction

Schistosomiasis (SCH) and soil-transmitted helminthiasis (STH) are among the most prevalent neglected tropical diseases (NTDs), disproportionately affecting impoverished communities in sub-Saharan Africa [1, 2]. SCH, caused by *Schistosoma* species (primarily *S. haematobium* in Nigeria), leads to urinary tract damage, haematuria, anaemia, and increased HIV susceptibility, with over 240 million global cases [3]. STH, including *Ascaris lumbricoides*, *Trichuris trichiura,* and hookworms, causes malnutrition, growth stunting, and cognitive impairment, infecting 1.5 billion people worldwide [4].

Nigeria has been at the forefront of the African region, with 134 million people at risk of SCH and widespread STH transmission [5]. The WHO NTD Roadmap 2021–2030 targets both diseases for elimination as a public health problem (EPHP) for SCH (<1% prevalence in SAC) and STH (<2% moderate/heavy intensity) by 2030, with impact assessments to be done after 3–5 MDA rounds [6].

Earlier baseline study in Akwa Ibom State was conducted in all the 31 LGAs. This study is focussing on the 26 LGAs where the recent impact survey was conducted after 5 effective rounds of MDA. The baseline surveys (2009-2017) in Akwa Ibom State revealed a high level of STH endemicity (25.1 - 91.4%), compared to a low level of SCH prevalence (0 - 1.2%) [7]. The Federal Ministry of Health (FMOH), in collaboration with the Akwa Ibom State Ministry of Health and support from Helen Keller Intl, responded with preventive chemotherapy (PC) by using albendazole/mebendazole to treat STH and praziquantel to treat SCH with >75% coverage every year or biannually. Between 2014 and 2023, a total of 5-8 effective therapeutic rounds for STH were administered in all 26 endemic Local Government Areas (LGAs) in Akwa Ibom State, Nigeria, in compliance with global best practice. These LGAs include; Abak, Eket, Essien Udim, Etim Ekpo, Etinan, Ibesikpo Asutan, Ibiono Ibom, Ika, Ikono, Ikot Ekpene, Ini, Itu, Mbo, Mkpat Enin, Nsit Atai, Nsit Ibom, Nsit Ubium, Obot Akara, Okobo, Oron, Oruk Anam, Udung Uko, Ukanafun, Uruan, Urue-Offong/Oruko, and Uyo. But in the case of SCH, between 2014 and 2023, the survey reflected low endemicity of the same LGAs of the state [8].

It is relevant to investigate the current status of the prevalence of these parasites in these LGAs in order to redefine their endemicity. This study assesses the impact of PC after 5–8 rounds on SCH and STH prevalence, intensity, and endemicity in 26 LGAs of Akwa Ibom State. The survey is part of the national and state NTD strategic plan to provide data needed for planning, implementation, and monitoring the progress of the SCH and STH control programme.

2. Methods

2.1. Study site

Akwa Ibom State is situated in the south-south region of Nigeria. Its location is between latitudes 4°32′1′′ and 5°33′1′′ North and longitudes 7°25′1′′ and 8°25′1′′ East. The state shares its borders with Rivers State on the East side, Cross River on the West, Abia State on the North and the Gulf of Guinea on the South. The study was conducted across 26 selected LGAs of Akwa Ibom State. These LGAs includes; Abak, Eket, Essien Udim, Etim Ekpo, Etinan, Ibesikpo Asutan, Ibiono Ibom, Ika, Ikono, Ikot Ekpene, Ini, Itu, Mbo, Mkpat Enin, Nsit Atai, Nsit-Ibom, Nsit Ubium, Obot Akara, Okobo, Oron, Oruk Anam, Udung Uko, Ukanafun, Uruan, Urue-Offiong/Oruko and Uyo (Figure 1).

2.2. Ethical considerations

The Akwa Ibom State Research Ethics Committee provided ethical approval. Community gatekeepers were provided with administrative clearance and letters of introduction. Parents or guardians of participating school-aged children (SAC) provided written informed consent and assent of children was obtained where necessary. It was voluntary and confidential.

2.3. Community sensitization/mobilization

The community mobilization was done by advocacy visits, local media and working with school authorities and community leaders. The research objectives, methods and voluntary nature of the study were clearly explained before the data collection.

2.4. Study design

The cross-sectional impact assessment at schools was carried out in October 2025 after 5-8 successful mass drug administrations (MDA). A two-stage sampling strategy consistent with recent WHO impact assessment guidelines was employed.

2.4.1 Selection of Primary Sampling Units (PSUs)

Purposive sampling was used to identify five wards per LGA, the baseline survey location and epidemiological relevance; this resulted in 130 PSUs in 26 LGAs.

2.4.2 Selection of Participants

A systematic sampling method was used to randomly select 52 SAC aged 8 years to 14 years (26 males and 26 females) in every PSU [9, 10]. In cases that the enrolment was more than the target numbers, random starting point and sample interval were used. In accordance with this criterion the total sample size of over 7,000 was aimed to be reached. There were however 6,431 valid samples that were analysed.

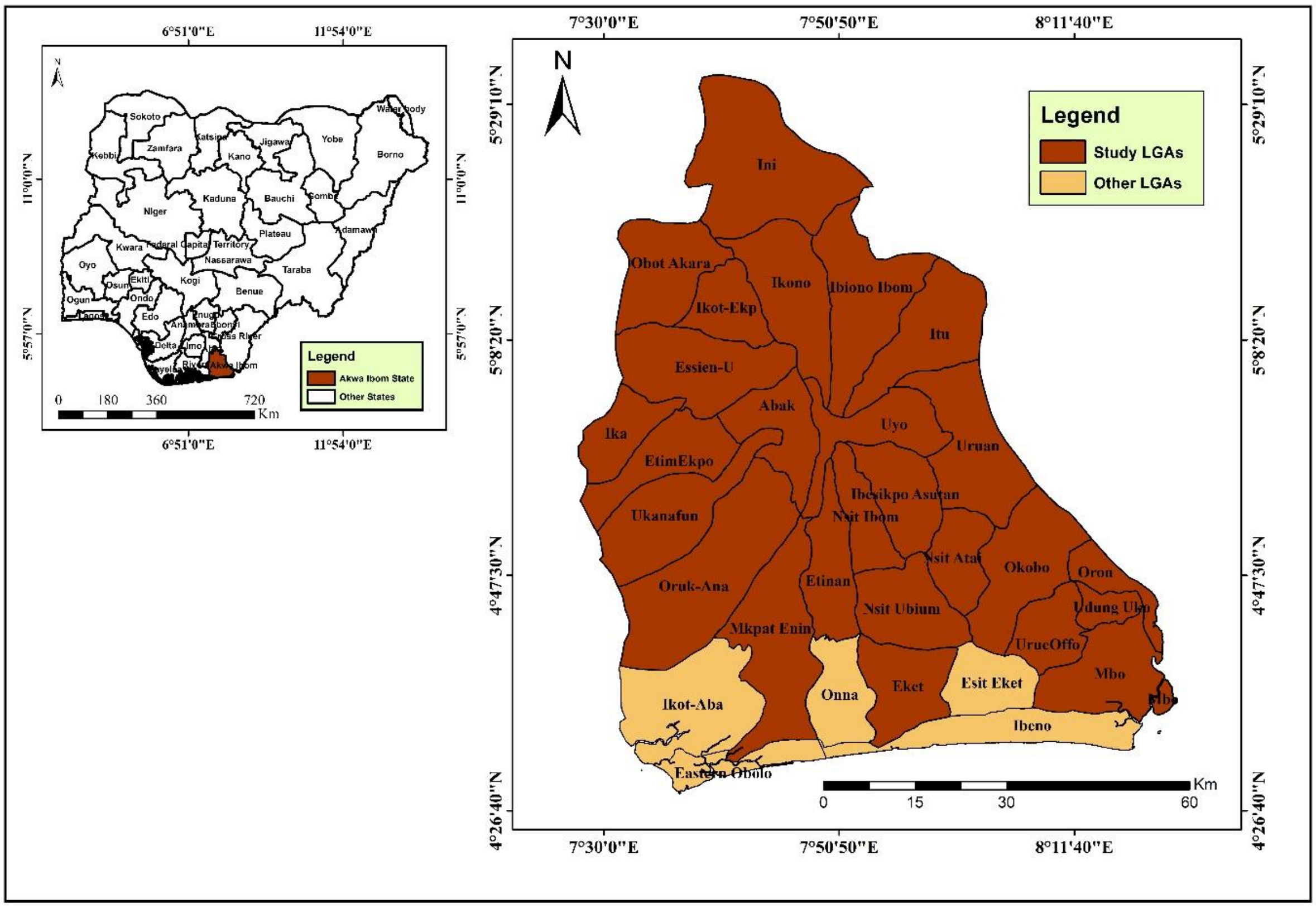

Figure 1. Map of the Study Area indicating LGAs surveyed.

2.5. Sample collection

School-aged children were grouped in terms of gender and age by a systematic random sampling procedure. Sampling intervals were applied in the situations where the enrolment was larger than the required (e.g., SI = total SAC/required; every n^{th} child sampled at a random starting point). All chosen children gave stool sample (Collected in labelled containers) and urine sample collected mid-morning (10:00-14:00 hours) so as to maximise the detection of *Schistosoma haematobium* egg excretion.

2.6. Laboratory examination of samples

Samples collected were transported to the laboratory for processing and examination. Parasite eggs in stools were analysed with the use of the Kato-Katz method on STH (Ascaris lumbricoides, *Trichuris trichiura*, and hookworm) and intestinal schistosome (*Schistosoma mansoni*) [11, 1]. Hookworm slides were read in less than 60 minutes to avoid the clearance of eggs. The counts of eggs were converted in terms of eggs per gram (epg) and categorized based on WHO intensity thresholds.

2.7. Electronic data collection

The electronic data collection was done using Android based devices linked to a cloud server. The results of school-level, participant-level, stool, and urine were recorded in four structured forms. The GPS coordinates of every PSU were noted. Data validation and backup procedures were monitored on a regular basis. Proper monitoring of the transmitted data was done during the study. Hardcopy backups were also maintained.

2.8. Data Analyses

Cleaning of data was done in Microsoft Excel. The prevalence between the categories of infection i.e. based on sex, age groups, and LGAs were determined through descriptive statistics. WHO thresholds were used to classify endemicity (low: <20% STH, under 10% SCH, moderate: 20-49; heavy: 50%+). The severity of the infections was determined based on WHO guidelines [12]. The 95% confidence intervals (CI) were used to calculate prevalence estimates. Chi-square (χ^2) analysis was performed to test associations. Bivariate logistic regression was used to estimate odds ratios (ORs) with 95% CI. The significant level was established at $p < 0.05$. Statistical analysis was conducted using Statistical Package for Social Sciences (SPSS) version 27.

3. Results

3.1. Participant characteristics

A total of 6,431 SAC were examined (52.7% males; 47.3% females). The majority (62.2%) were aged 8–10 years, while 37.8% were aged 11–14 years (Table 1).

3.2. Prevalence of SCH and STH infection in 26 LGAs of the State

A total of 6431 students participated in this survey, comprising of 52.7% males (3392 individuals) and 47.3% females (3039 individuals), with 62.2% (3998 individuals) in the age group of 8 to 10 years and 37.8% (2433 individuals) in the age group of 11 to 14 years.

In this survey, samples from all SAC revealed they were not infected with *Schistosoma mansoni* and *Schistosoma haematobium*, 0.0% prevalence was recorded in all the PSUs across the 26 LGAs studied (Table 2). An overall prevalence of 27.62% [95% C.I. = 26.54 – 28.72%] (1776 students) was recorded among SAC for STH in this survey, with parasite-specific prevalence of 15.38% [95% C.I. = 14.52 – 16.28%] for hookworms, 15.10% [95% C.I. = 14.24 – 15.99%] for *Ascaris lumbricoides,* and 1.52% [95% C.I. = 1.25 – 1.85%] for *Trichuris trichiura*. Co-infection was highest between *Ascaris lumbricoides* and hookworm (3.39%), and least between *Trichuris trichiura* and hookworm (0.47%) (Table 2).

The result of STH prevalence by LGA is reflected in the Table 3. Mbo (71.65%) had the highest prevalence, followed by Udung Uko (62.04%), and the least prevalence was recorded at Ika LGA (4.92%). Comparison of the prevalence among the LGAs was statistically significant ($p < 0.05$%) (Figure 2, Table 3). *Ascaris lumbricoides* infection was highest at Mbo LGA (67.0%, 175 children), followed by Udung Uko LGA (57.7%, 158 children) and Okobo (40.8%, 91 children), while the lowest prevalence was at Ika LGA (1.6%, 4 children). Prevalence of *Ascaris lumbricoides* was statistically significant ($p<0.001$) across the LGAs (Table 4, Figure 3). *Trichuris trichiura* prevalence was highest at Uruan LGA (9.3%, 23 children), followed by Mbo LGA (9.2%, 24 children). Prevalence was also statistically significant across the LGA ($p <0.001$) (Table 4, Figure 4). Hookworms infection was highest at Ini LGA (38.3%, 103 children), followed by Nsit Ubium LGA (30.8%, 76 children), while the lowest prevalence was recorded at Ika LGA (3.3%, 8 children), the variation in prevalence was statistically significant across the LGAs (Table 4, Figure 5).

Age specific prevalence revealed that school children in the age group of 11-14 years, 31.77% [95% C.I. = 29.95 – 33.64%] had higher prevalence than those in the age group of 8 to 10 years [95% C.I. = 23.77 – 26.46%] (Table 5). Comparison between the age groups was statistically significant ($p < 0.05$). Sex specific prevalence revealed a higher prevalence in males [29.30%, 95% C.I = 27.80 – 30.86%] than in females [25.73%, 95% C.I. = 24.24 – 27.35%], comparison of prevalence between sexes was statistically significant ($p < 0.05$) (Table 5).

Table 1. Demographics of Study Participants

Variables	N = 6431	%
**Gender**		
Male	3392	52.7
Female	3039	47.3
**Age**		
8-10	3998	62.2
11-14	2433	37.8

Table 2. Prevalence of *Schistosoma* spp. and STH by Species

**Prevalence**	**NE**	**NI (%)**	**95% C.I.**
***Schistosoma* spp**	6431	0 (0.00)	NA
Schistosoma mansoni	6431	0 (0.00)	NA
Schistosoma haematobium	6431	0 (0.00)	NA
**STH**	6431	1776 (27.62)	26.54 – 28.72
Ascaris lumbricoides	6431	971 (15.10)	14.24 – 15.99
Trichuris trichiura	6431	98 (1.52)	1.25 – 1.85
Hookworm	6431	989 (15.38)	14.52 – 16.28
**Co-infection**			
Ascaris spp + *Trichuris spp*	6431	55 (0.86)	0.66 – 1.11
Ascaris spp + Hookworm	6431	218 (3.39)	2.97 – 3.86
Trichuris spp + Hookworm	6431	30 (0.47)	0.32 – 0.67
Ascaris spp + *Trichuris spp* + Hookworm	6431	21 (0.33)	0.21 – 0.50

NE – Number Examined, NI – Number Infected, NA – Not Applicable.

Table 3. Prevalence of *Schistosoma* spp. and STH by LGA

**LGA**	**NE**	**NI (%) with *Schistosoma* spp.**	**NI (%) with STH**	**95% C.I. of STH**
Abak	249	0 (0.00)	39 (15.66)	11.56 - 20.56
Eket	260	0 (0.00)	52 (20.00)	15.49 - 25.18
Essien Udim	252	0 (0.00)	61 (24.21)	19.23 - 29.77
Etim Ekpo	225	0 (0.00)	23 (10.22)	6.77 - 14.69
Etinan	234	0 (0.00)	26 (11.11)	7.57 - 15.61
Ibesikpo Asutan	237	0 (0.00)	59 (24.89)	19.72 - 30.68
Ibiono Ibom	226	0 (0.00)	45 (19.91)	15.11 - 25.48
Ika	244	0 (0.00)	12 (4.92)	2.72 - 8.18
Ikono	232	0 (0.00)	26 (11.21)	7.63 - 15.74
Ikot Ekpene	239	0 (0.00)	25 (10.46)	7.06 - 14.81
Ini	269	0 (0.00)	108 (40.15)	34.42 - 46.09
Itu	259	0 (0.00)	42 (16.22)	12.11 - 21.07
Mbo	261	0 (0.00)	187 (71.65)	65.96 - 76.85
Mkpat Enin	258	0 (0.00)	84 (32.56)	27.06 - 38.45
Nsit Atai	253	0 (0.00)	82 (32.41)	26.87 - 38.35
Nsit Ibom	246	0 (0.00)	42 (17.07)	12.77 - 22.15
Nsit Ubium	247	0 (0.00)	83 (33.60)	27.93 - 39.66
Obot Akara	259	0 (0.00)	60 (23.17)	18.34 - 28.58
Okobo	223	0 (0.00)	118 (52.91)	46.36 - 59.39
Oron	252	0 (0.00)	107 (42.46)	36.47 - 48.62
Oruk Anam	239	0 (0.00)	54 (22.59)	17.64 - 28.21
Udung Uko	274	0 (0.00)	170 (62.04)	56.20 - 67.64
Ukanafun	234	0 (0.00)	44 (18.80)	14.20 - 24.18
Uruan	246	0 (0.00)	102 (41.46)	35.44 - 47.69
Urueoffong/Oruko	261	0 (0.00)	93 (35.63)	30.01 - 41.58
Uyo	252	0 (0.00)	32 (12.70)	9.02 - 17.23
**Total**	**6431**	**0 (0.00)**	**1776 (27.62)**	**26.53 - 28.72**
χ^2			881.31	
df			25	
p Value			<0.001	

NE – Number Examined, NI – Number Infected.

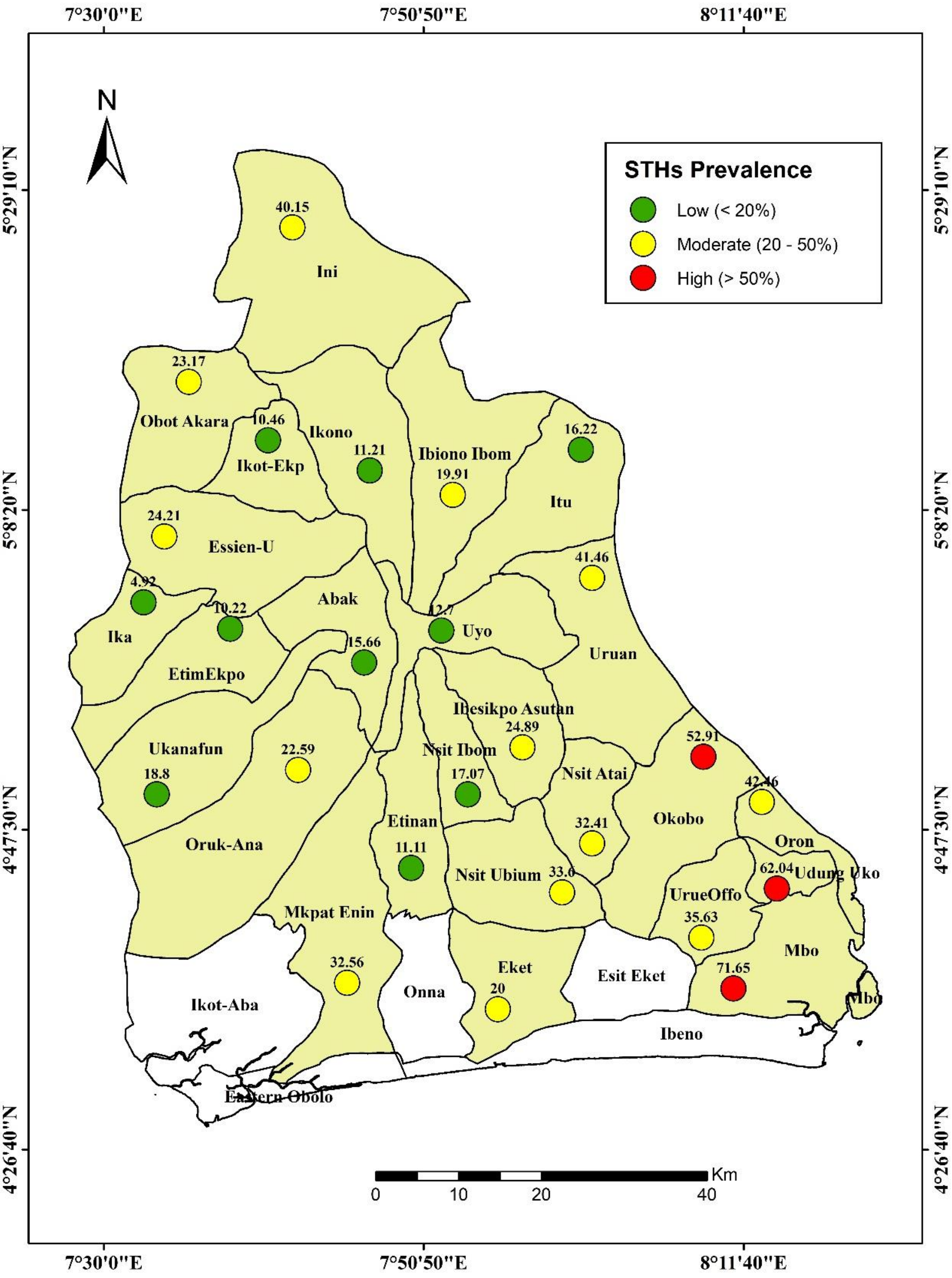

Figure 2. Prevalence of Soil-Transmitted Helminths across the 26 Endemic LGAs of Akwa Ibom State, Nigeria

Table 4. Prevalence of *Ascaris lumbricoides*, *Trichuris trichiura* and Hookworms by LGA

**LGA**	**NE**	***Ascaris lumbricoides* (%)**	***Trichuris trichiura* (%)**	**Hookworms (%)**
Abak	249	12 (4.8)	1 (0.4)	30 (12.0)
Eket	260	31 (11.9)	4 (1.5)	23 (8.8)
Essien Udim	252	9 (3.6)	1 (0.4)	57 (22.6)
Etim Ekpo	225	10 (4.4)	1 (0.4)	18 (8.0)
Etinan	234	4 (1.7)	0 (0.0)	24 (10.3)
Ibesikpo Asutan	237	20 (8.4)	3 (1.3)	44 (18.6)
Ibiono Ibom	226	13 (5.8)	1 (0.4)	33 (14.6)
Ika	244	4 (1.6)	0 (0.0)	8 (3.3)
Ikono	232	8 (3.4)	0 (0.0)	19 (8.2)
Ikot Ekpene	239	7 (2.9)	1 (0.4)	22 (9.2)
Ini	269	7 (2.6)	2 (0.7)	103 (38.3)
Itu	259	17 (6.6)	5 (1.9)	27 (10.4)
Mbo	261	175 (67.0)	24 (9.2)	33 (12.6)
Mkpat Enin	258	49 (19.0)	1 (0.4)	55 (21.3)
Nsit Atai	253	17 (6.7)	1 (0.4)	71 (28.1)
Nsit Ibom	246	10 (4.1)	0 (0.0)	35 (14.2)
Nsit Ubium	247	13 (5.3)	0 (0.0)	76 (30.8)
Obot Akara	259	6 (2.3)	1 (0.4)	54 (20.8)
Okobo	223	91 (40.8)	8 (3.6)	46 (20.6)
Oron	252	92 (36.5)	9 (3.6)	29 (11.5)
Oruk Anam	239	34 (14.2)	1 (0.4)	29 (12.1)
Udung Uko	274	158 (57.7)	6 (2.2)	27 (9.9)
Ukanafun	234	34 (14.5)	0 (0.0)	11 (4.7)
Uruan	246	60 (24.4)	23 (9.3)	55 (22.4)
Urueoffong/Oruko	261	76 (29.1)	4 (1.5)	40 (15.3)
Uyo	252	14 (5.6)	1 (0.4)	20 (7.9)
**Total**	**6431**	**971 (15.1)**	**98 (1.5)**	**989 (15.4)**
χ^2		1567.56	261.008	341.296
df		25	25	26
p Value		<0.001*	<0.001*	<0.001*

NE – Number Examined.

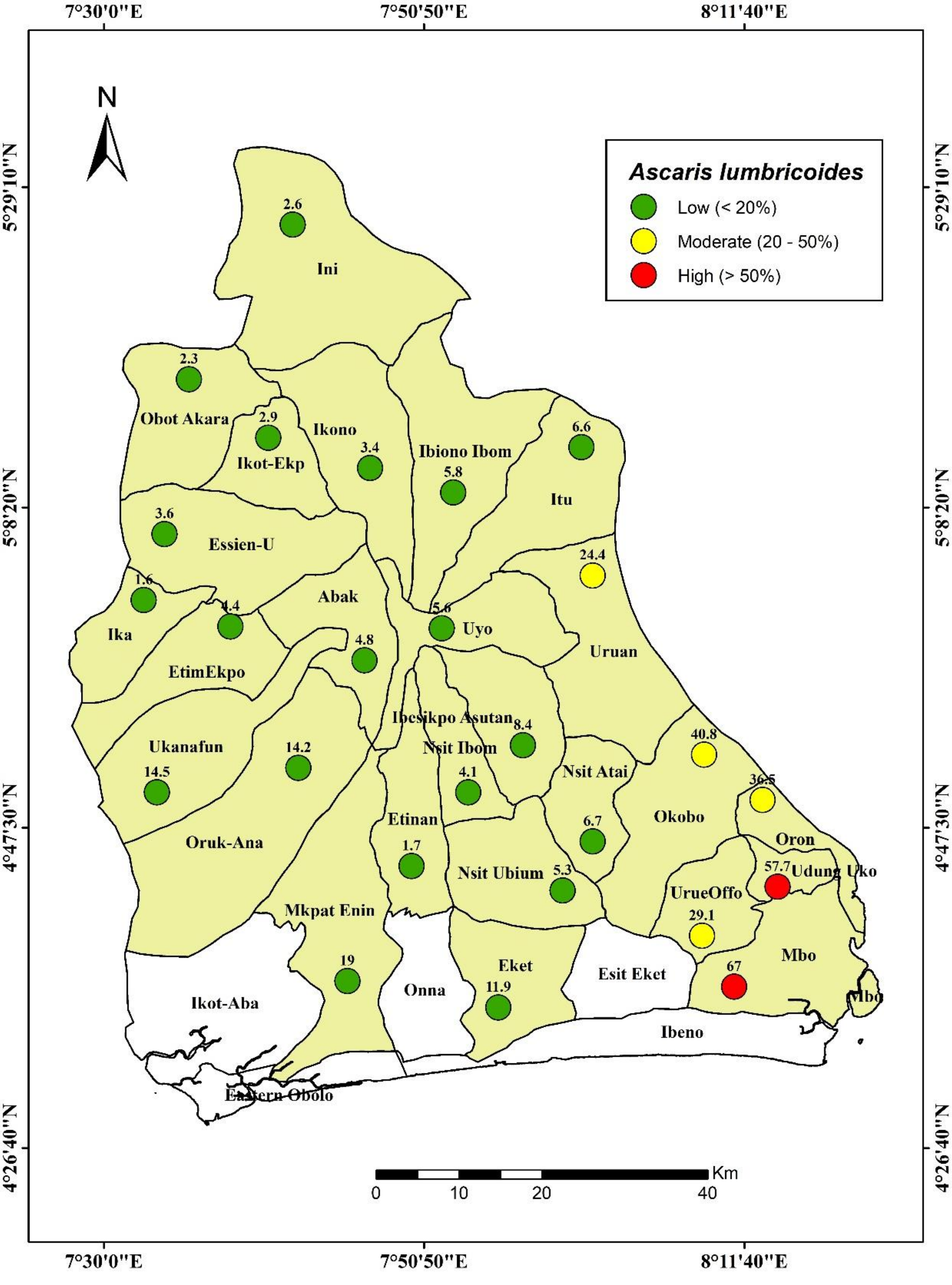

Figure 3. Prevalence of *Ascaris lumbricoides* across the 26 Endemic LGAs of Akwa Ibom State, Nigeria.

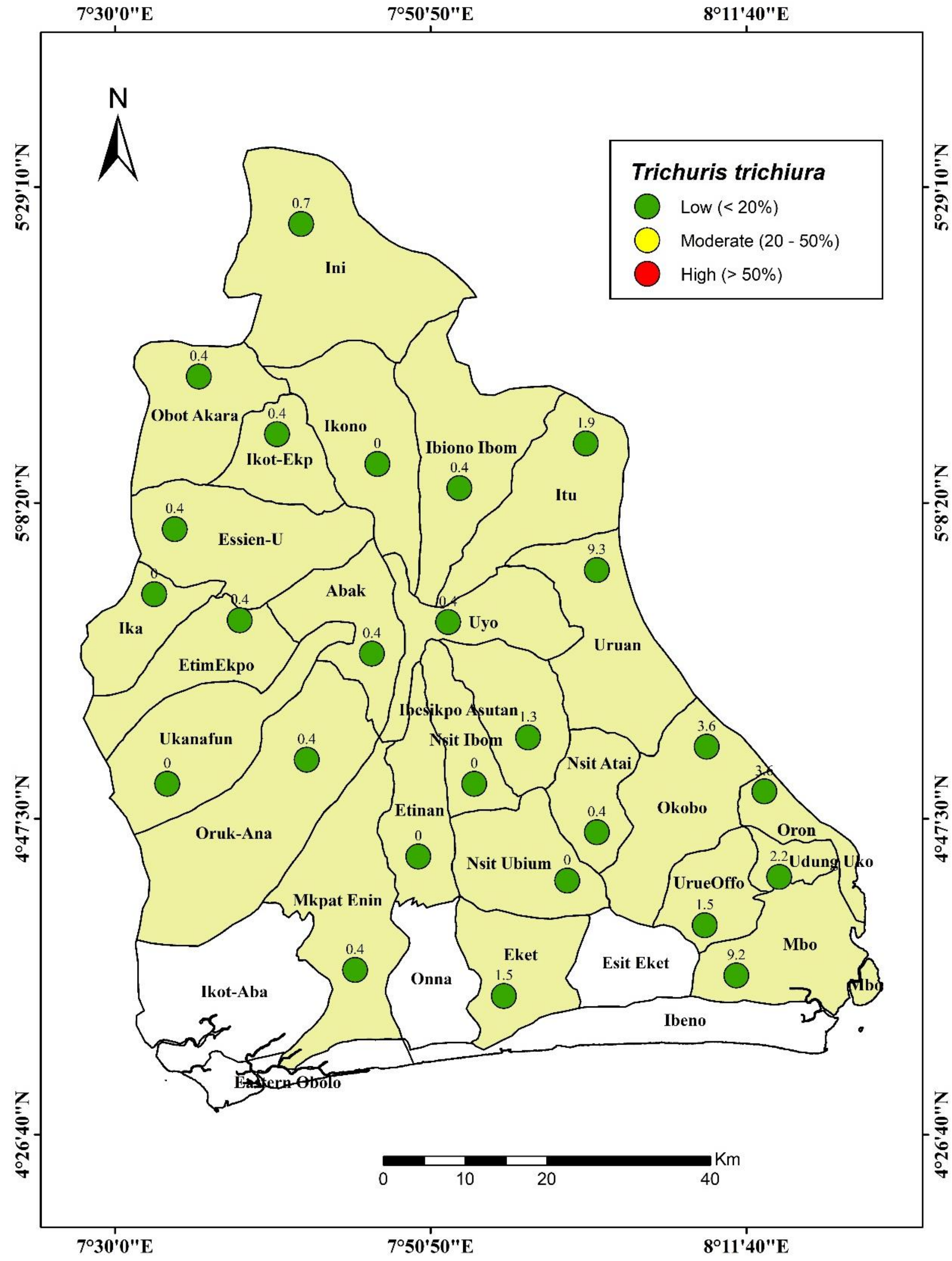

Figure 4. Prevalence of *Trichuris trichiura* across the 26 Endemic LGAs of Akwa Ibom State, Nigeria.

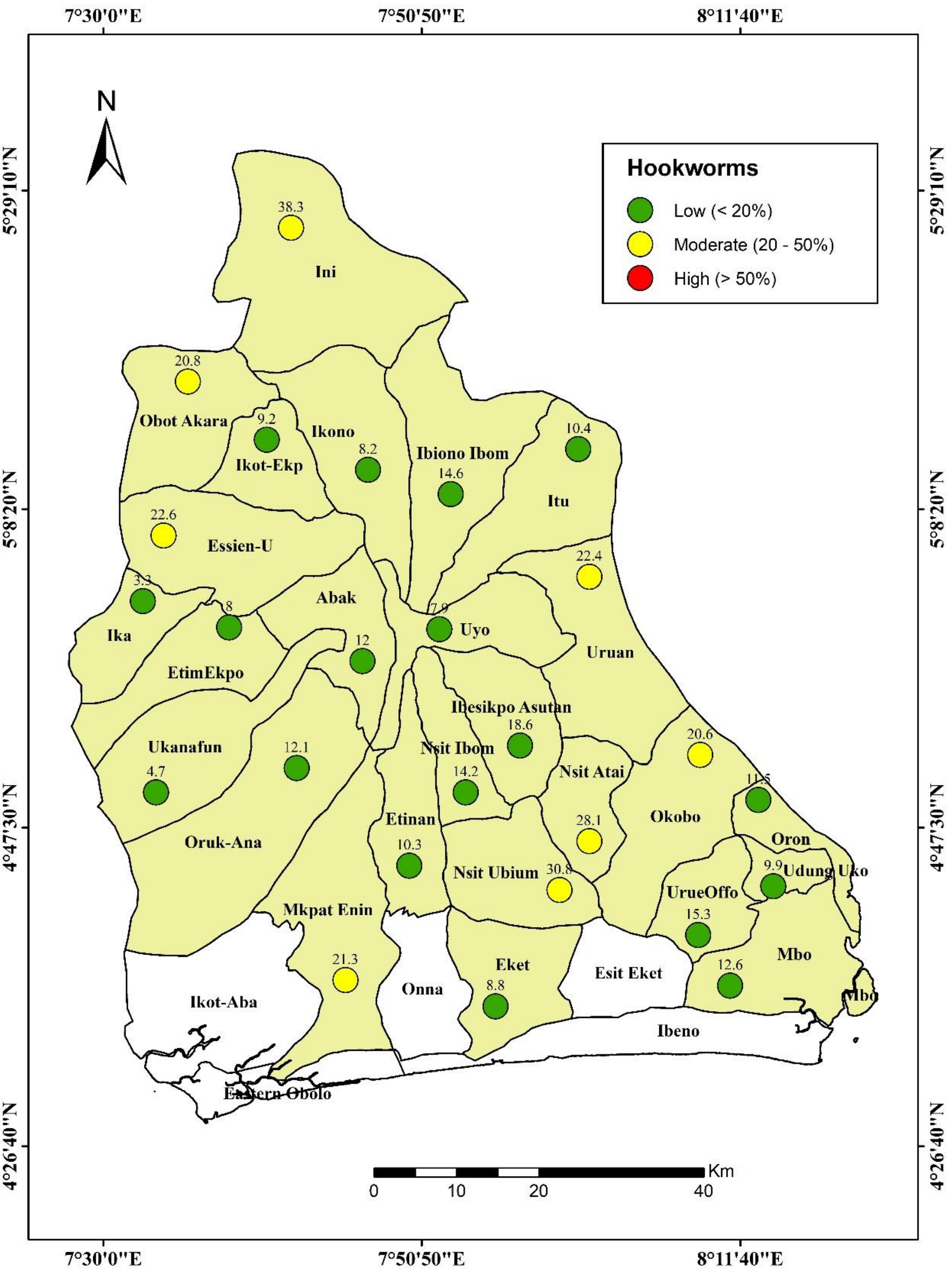

Figure 5. Prevalence of Hookworms across the 26 Endemic LGAs of Akwa Ibom State, Nigeria.

Table 5. Overall Prevalence of *Schistosoma* spp. and STH by Age Groups and Sex

Age and Sex	NE	NI (%) with *Schistosoma* spp.	NI (%) with STH	95% C.I. of STH
**Age Groups (Years)**				
7-10	3998	0 (0.00)	1003 (25.09)	23.77 - 26.46
11-14	2433	0 (0.00)	773 (31.77)	29.95 - 33.65
	**6431**	**0 (0.00)**	**1776 (27.62)**	**26.54 - 28.72**
χ^2		NA	33.804	
df		NA	1	
P Value		Na	<0.001*	
**Sex**				
Male	3392	0 (0.00)	994 (29.30)	27.80 - 30.86
Female	3039	0 (0.00)	782 (25.73)	24.24 - 27.35
	**6431**	**0 (0.00)**	**1776 (27.62)**	**26.54 - 28.72**
χ^2		NA	10.232	
df		NA	1	
P Value		NA	<0.001*	

NE – Number Examined, NI – Number Infected, NA – Not Applicable.

3.3. STH Infection Load Estimation

A total of 98.87% (1756 individuals) of infected school children had light intensity infection, and 1.13% (20 individuals) had moderate intensity infection. High intensity infected was not recorded across the 26 LGAs (Table 6).

3.4. Comparison between Baseline and Current Prevalence of STH in Akwa Ibom State

An overall prevalence of 27.62% was recorded in the current survey of October 2025, with prevalence (ranges: 4.92% to 71.65%) compared to baseline prevalence of 57.77% (range: 25.1% to 91.39%) for Akwa Ibom State, with a 30.15% reduction in prevalence. Across the 26 LGAs surveyed, there was a reduction in prevalence compared to the baseline. Although some LGAs recorded a significant reduction than others, Abak LGA reported the highest significant reduction in prevalence with over 56.54% reduction (15.66% current, 72.2% baseline), followed by Itu LGA with 43.25% reduction (16.22% current, 59.47% baseline), while Mbo LGA has the least reduction in prevalence with 11.1% reduction (71.65% current, 82.75% baseline) (Figure 6, Table 7).

3.5. Risk Factors of STH and Infection Status

The study also examined the socio-demographic characteristics and WASH factors in relation to the infection status. The risk of acquiring STH infections was significantly associated with sex, age group of school children, location of toilet facility, when and where children urinate and defecate in schools (Table 7). According to the sex, male school children had a higher likelihood of being infected with STH (OR = 1.196, 95% CI: 1.072 – 1.335). Children in the age group of 11 to 14 years (OR = 1.391, 95% C.I.: 1.244 – 1.554) are at higher risk of being infected with STH. School children that use bush (OR = 1.706 (95% C.I. = 1.155 – 2.520) and dry pit (OR = 1.911, 95% C.I. = 1.589 – 2.299) had higher likelihood of being infected with STH. The location of toilet facility is a major factor influencing the risk of being infected with STH, school children that live where the toilet facility is located outside the school environment had higher likelihood of being infected with STH (OR = 1.218, 95% C.I. = 1.089 – 1.361). School children that live in environment where urination (OR = 1.213, 95% C.I.: 1.084 – 1.358) and defecation (OR = 1.225, 95% C.I. = 1.080 – 1.388) is carried out around the school environment are at higher risk of being infected with STH (Table 7).

Table 6. Intensity of STH Infection

LGA	NE	NI	Light Infection (%) 1–999 epg	Moderate Infection (%) 1000–9999 epg	Heavy Infection (%) ≥10,000 epg
Abak	249	39	37 (94.87)	2 (5.13)	-
Eket	260	52	52 (100.00)	-	-
Essien Udim	252	61	60 (98.36)	1 (1.64)	-
Etim Ekpo	225	23	23 (100.00)	-	-
Etinan	234	26	26 (100.00)	-	-
Ibesikpo Asutan	237	59	57 (96.61)	2 (3.39)	-
Ibiono Ibom	226	45	45 (100.00)	-	-
Ika	244	12	12 (100.00)	-	-
Ikono	232	26	26 (100.00)	-	-
Ikot Ekpene	239	25	24 (96.00)	1 (4.00)	-
Ini	269	108	108 (100.00)	-	-
Itu	259	42	42 (100.00)	-	-
Mbo	261	187	186 (99.47)	1 (0.53)	-
Mkpat Enin	258	84	83 (98.81)	1 (1.19)	-
Nsit Atai	253	82	82 (100.00)	-	-
Nsit Ibom	246	42	42 (100.00)	-	-
Nsit Ubium	247	83	83 (100.00)	-	-
Obot Akara	259	60	60 (100.00)	-	-
Okobo	223	118	118 (100.00)	-	-
Oron	252	107	106 (99.07)	1 (0.93)	-
Oruk Anam	239	54	54 (100.00)	-	-
Udung Uko	274	170	166 (97.65)	4 (2.35)	-
Ukanafun	234	44	44 (100.00)	-	-
Uruan	246	102	96 (94.12)	6 (5.88)	-
Urueoffong/Oruko	261	93	93 (100.00)	-	-
Uyo	252	32	31 (96.88)	1 (3.13)	-
Total	**6431**	**1776**	**1756 (98.87)**	**20 (1.13)**	**0 (0.00)**

NE – Number Examined, NI – Number Infected.

Table 7. Comparison between Baseline and Current Prevalence of STH in Akwa Ibom State

LGAs	Baseline (%)	Current (%)	Difference (%) = Baseline - Current
Abak	72.2	15.66	56.54
Eket	57.14	20	37.14
Essien Udim	48.4	24.21	24.19
Etim Ekpo	25.1	10.22	14.88
Etinan	47.69	11.11	36.58
Ibesikpo Asutan	64.03	24.89	39.14
Ibiono Ibom	56.15	19.91	36.24
Ika	41.18	4.92	36.26
Ikono	29.92	11.21	18.71
Ikot Ekpene	46.22	10.46	35.76
Ini	65.35	40.15	25.2
Itu	59.47	16.22	43.25
Mbo	82.75	71.65	11.1
Mkpat Enin	63.35	32.56	30.79
Nsit Atai	71.26	32.41	38.85
Nsit Ibom	39.29	17.07	22.22
Nsit Ubium	62.93	33.6	29.33
Obot Akara	59.13	23.17	35.96
Okobo	80.93	52.91	28.02
Oron	66.53	42.46	24.07
Oruk Anam	55.54	22.59	32.95
Udung Uko	91.39	62.04	29.35
Ukanafun	36.86	18.8	18.06
Uruan	67.8	41.46	26.34
Urueoffong/Oruko	75.59	35.63	39.96
Uyo	35.94	12.7	23.24
**Total**	**57.77**	**27.62**	**30.15**
Range	25.1 to 91.39	4.92 to 71.65	

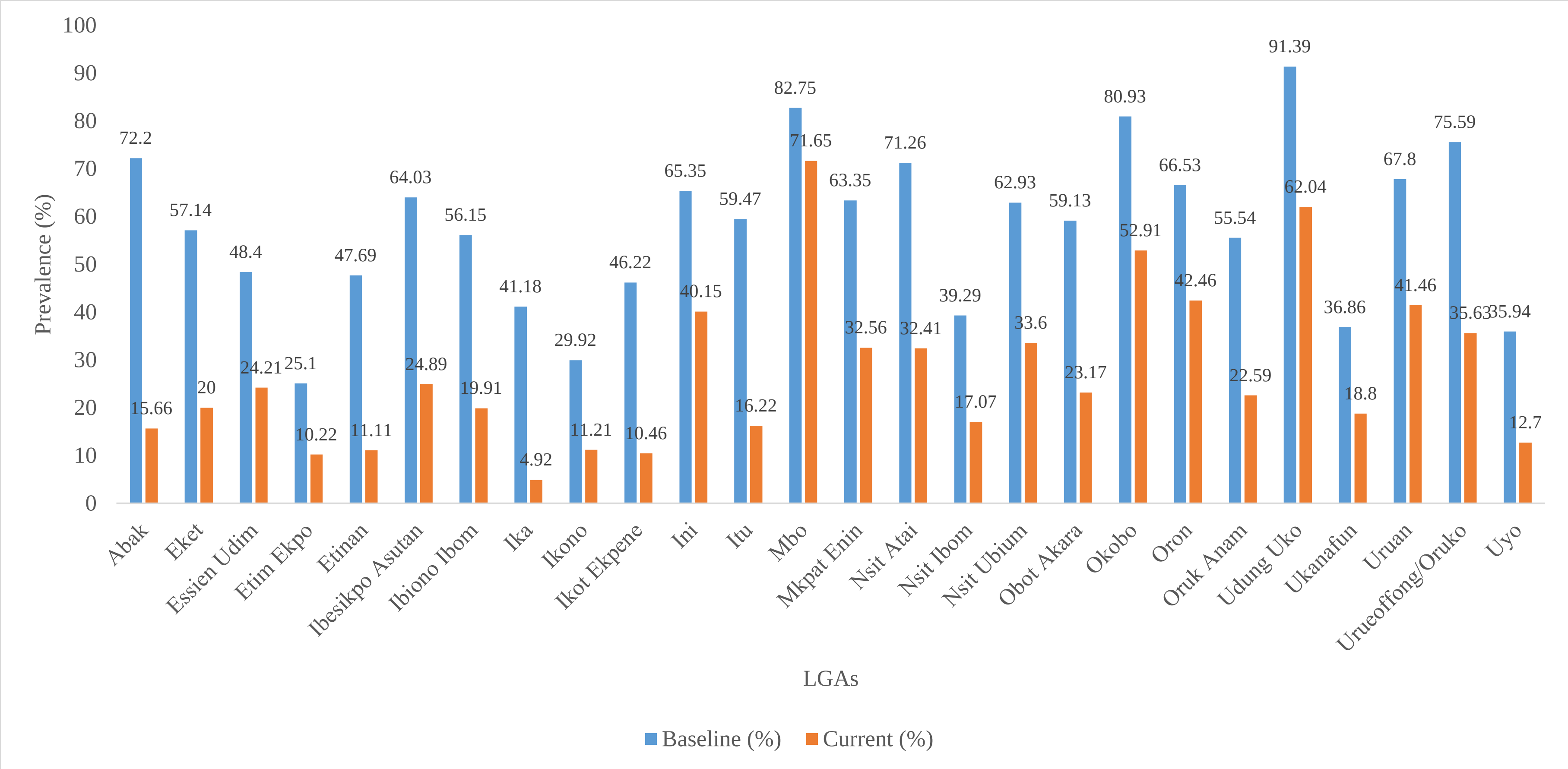

Figure 6. Comparison between Baseline and Current Prevalence of Soil Transmitted Helminths in 26 Endemic LGAs of Akwa Ibom State, Nigeria.

Table 7. Bivariate Analysis of Risk Factors Associated with STH Infection in the Study Area

Characteristics	NE	NI (%)	χ^2	*p*	OR (95% C.I.)
**Sex**					
Male	3392	994 (29.30)	10.232	0.001*	1.196 (1.072 – 1.335)*
Female	3039	782 (25.73)			1
**Age (years)**					
8 – 10	3998	1003 (25.09)	33.804	< 0.001*	1
11 - 14	2433	773 (31.77)			1.391 (1.244 – 1.554)*
**Hand washing after defecation**					
No	1040	294 (28.27)	0.265	0.607ns	1.040 (0.897 – 1.205)
Yes	5391	1482 (27.49)			1
**Use of soap and water to wash hands**					
No	1790	388 (21.68)	2.620	0.106ns	0.897 (0.787 – 1.023)
Yes	4641	1094 (23.57)			1
**Toilet facility used by household**					
Bush	163	43 (26.38)	25.644	< 0.001*	1.706 (1.155 – 2.520)*
Bucket	18	4 (22.22)			1.361 (0.442 – 4.190)
Composting	5	0 (0.00)			NA
Container	2	1 (50.00)			4.762 (0.296 – 76.553)
Dry pit	5506	1577 (28.64)			1.911 (1.589 – 2.299)*
Flush/WC	734	151 (41.14)			1
Hanging	3	0 (0.00)			NA
**When you are at school, where do you usually go to urinate?**					
Around the school compound	2561	786 (30.69)	62.977	< 0.001*	1.213 (1.084 – 1.358)*
I wait/hold	40	16 (40.00)			1.827 (0.966 – 3.454)
In a nearby home or bush	282	27 (9.58)			0.290 (0.194 – 0.434)
In the school toilet	3508	938 (26.74)			1
Outside the school compound	40	9 (22.50)			0.795 (0.377 – 1.677)
**When you are at school, where do you usually go to defecate?**					
Around the school compound	1758	541 (30.77)	49.754	< 0.001*	1.225 (1.080 – 1.388)*
I wait/hold	192	78 (40.63)			1.885 (1.400 – 2.537)*
In a nearby home or bush	615	118 (19.19)			0.653 (0.527 – 0.808)
In the school toilet	3612	962 (26.63)			1
Outside the school compound	254	77 (30.32)			1.198 (0.908 – 1.582)
**What is the main source of drinking water for members of your household?**					
Hand Pump	689	258 (37.45)	135.009	<0.001*	1
Piped into private dwelling	119	23 (19.33)			0.400 (0.248 – 0.647)
Piped into private yard	211	42 (19.91)			0.415 (0.286 – 0.602)
Protected dug well	2033	558 (27.45)			0.632 (0.527 – 0.759)
Protected spring	680	157 (23.09)			0.502 (0.396 – 0.635)
Public-piped water	1075	211 (19.63)			0.408 (0.329 – 0.506)

Rainwater	338	98 (29.00)			0.682 (0.515 – 0.904)
Sachet	11	4 (36.36)			0.955 (0.277 – 3.293)
Surface water	825	313 (37.94)			1.021 (0.829 – 1.258)
Tanker truck	2	1 (50.00)			1.671 (0.104 – 26.824)
Unprotected dug well	16	7 (43.75)			1.299 (0.478 – 3.531)
Unprotected spring	432	104 (24.07)			0.530 (0.405 – 0.693)
**Taken deworming tablet before?**					
No	240	52 (21.70)	4.415	0.036*	0.717 (0.525 – 0.979)
Yes	6191	1724 (27.80)			1
**Wearing any footwear (sandals, shoes, slippers e.t.c.)**					
No	64	11 (17.19)	3.517	0.061ns	0.541 (0.282 – 1.038)
Yes	6367	1765 (27.72)			1

ns – Not significant at p>0.05, * - Significant at p≤0.05, NA – Not applicable.

4. Discussion

The WHO targeted the elimination of schistosomiasis and soil-transmitted helminthiasis by 2030. In view of this, states that have conducted several rounds of chemotherapy need to assess the status of these diseases through conducting impact assessments to review the control strategies. Previously, impact assessment was conducted in 31 STH endemic and 15 SCH moderately endemic LGAs of Akwa Ibom State after receiving minimum of 5 effective rounds of MDA between 2015 to 2024. In 2024, impact assessment was conducted in 7 LGAs of which 5 were co-endemic for SCH and STH.

The current impact assessment was carried in October 2025, to understand the current distribution of schistosomiasis and soil-transmitted helminthiasis across 26 LGAs. The observation from the current survey carried out in 26 LGAs of Akwa Ibom State revealed 0.0% prevalence of *Schistosoma* spp. and 27.62% overall prevalence of STH, indicating a 30.15% significant reduction in the overall prevalence of STH infection compared to the baseline survey of 2014. The state achieved effective programmatic action against schistosomiasis across the 26 LGAs, with all the wards visited having a prevalence threshold below the WHO recommendation. The state also recorded significant achievement in the reduction of prevalence of STH with a 30% reduction in overall prevalence, and varying reduction in prevalence across the LGAs. However, for STH, no LGA has met the WHO treatment threshold of less than 2% for stopping MDA, which mandates the need to intensify effort in the control of STH [13]. Despite the significant reduction in the prevalence of STH, factors such as lack of drugs, delayed supply chain, inability to conduct 2 rounds of MDA in some LGAs requiring such regimen must have affected the high prevalence observed in some LGAs.

The persistent dominance of hookworms (15.38%) is a significant public health concern, particularly due to its direct association with anaemia and cognitive impairment in children. The observed statistically significant difference in infection rates between males and females suggests differential exposure to environmental risk factors, which must inform targeted behavioural change communication strategies. A similar observation was reported in a previous study in Plateau State [14]. Children in the age group of 11 to 14 years were affected more, indicating exposure to more high-risk environments and behaviours associated with that age. A study reported the same pattern for that age group of 11 to 14 years in Ondo State, Nigeria [1].

The survey confirms heterogeneity in prevalence with significant variation in the distribution of the parasites across the LGAs, which indicates the need to identify key elements resulting to the spatial variation in prevalence across the state, a programmatic challenge that should be tailored alongside chemotherapy for an intensified and concerted intervention package. Such packages include identifying non-functional basic water, sanitation and hygiene infrastructures, improvement in access and use of WASH through behavioural change, health and nutrition education, among other. The WHO recent guidelines emphasize on the need for improved access to water, sanitation and hygiene infrastructures [9].

Availability of toilets in schools, and the type of toilet available in the school environment for defecation, was a significant risk factor influencing the prevalence of STH in Akwa Ibom State. Also, clean-up behaviours in schools were found to be a significant contributor of infection. Children who urinated or defecated freely around the school compound had the highest rates of infection. This observation implies that a lack of school sanitation facilities, poor maintenance, or overcrowding could be making a significant contribution to the transmission. Enhancement of school WASH and better behaviour change communication among the pupils are still important actions that should be incorporated into the control programs [15]. Failure and ineffective performance of the control program caused by lack of access to WASH have been reported in different countries [16, 17, 18, 19].

In general, this assessment confirms that the transmission of soil-transmitted helminth parasites in these communities is influenced by the combination of environmental, behavioural, and infrastructural-related elements. The responses to the determinants must be integrated in health, education, water, hygiene and sanitation. The integration and utilization of the novel diagnostic technologies, such as the AiDx digital microscope, has proven to be a good complement to microscopy in accelerating laboratory analysis time and enhancing data throughput for time-sensitive programmatic decisions [20, 21, 22]. (Jacob *et al.*, 2025).

5. Conclusion

Preventive chemotherapy (PC) program for schistosomiasis and soil-transmitted helminthiasis (STH) in Akwa Ibom state has proven highly effective for schistosomiasis with 0.0% prevalence compared to baseline of 0.0 to 1.2% but partly effective for soil-transmitted helminthiasis with overall prevalence of 27.62% (ranges from 4.92 – 71.65%) compared to baseline of 25.1 to 91.4%. The survey revealed persistence in the prevalence of STH, with prevalence observed in all study sites above the 2% WHO recommended threshold for stopping treatment. This survey emphasizes the need for sustained treatment and control programs coupled with improved water, sanitation and hygiene.

Acknowledgements

We thank the Federal Ministry of Health and Social Welfare (FMOHSW), Akwa Ibom State Ministry of Health, Helen Keller Intl, The END Fund and the HIS for their support during this study.

Author contributions

Conceptualization: Kenneth Nnamdi Opara, Aliyu Mohammed, Imabong O. Umah

Data curation: Aliyu Mohammed, Imabong O. Umah

Formal analysis: Clement Ameh Yaro, Nnamso Williams Ikpeida

Investigation: Kenneth Nnamdi Opara, Clement Ameh Yaro, Nnamso Williams Ikpeida, Aliyu Mohammed, Hadiza Joy Marcu, Kingsley Azo, Salisu Ahmad, Esther Sunday Okon, Ellie Leaning, Moses Aderogba, Louise-Makau Barasa, Antia Etop, Imeh Udo Sylvester, Fatai Oyediran, Imabong O. Umah, Solomon M. Jacob, Gideon U. Ntuen, Veronica S. Augustine, Halima Toro

Methodology: Aliyu Mohammed, Imabong O. Umah

Supervision: Kenneth Nnamdi Opara, Aliyu Mohammed, Imabong O. Umah

Validation: Kenneth Nnamdi Opara, Aliyu Mohammed

Visualization: Clement Ameh Yaro

Writing – original draft: Kenneth Nnamdi Opara, Clement Ameh Yaro, Nnamso Williams Ikpeida

Writing – review and editing: Aliyu Mohammed, Imabong O. Umah

References

1. Mogaji HO, Olamiju FO, Oyinlola F, Achu I, Adekunle ON, Udofia LE, *et al*. Prevalence, intensity and risk factors of soil-transmitted helminthiasis after five effective rounds of preventive chemotherapy across three implementation units in Ondo State, Nigeria. PLoS Negl Trop Dis. 2024;19(1):e0012533. https://doi.org/10.1371/journal.pntd.0012533
2. Stothard JR, Chitsulo L, Kristensen TK, Utzinger J. Control of schistosomiasis in sub-Saharan Africa: progress made, new opportunities and remaining challenges. *Lancet* Parasitol. 2009;136(13):1665-75. https://doi.org/10.1017/s0031182009991272
3. WHO. Schistosomiasis and soil-transmitted helminthiases: progress report, 2020. Weekly Epid Rec. 2021;48(96):585-596. Available from https://www.who.int/publications/i/item/who-wer9648-585-595
4. Spencer S. The Global Burden of Disease 2010 Study. Glob Cardiol Sci Pract. 2013;13(2):115-126. https://doi.org/10.5339/gcsp.2013.15
5. Oluwole AS, Ekpo UF, Nebe OJ, Akpan NM, Jacob SM, Amazigo UV *et al*. The new WHO guideline for control and elimination of human schistosomiasis: implications for the Schistosomiasis Elimination Programme in Nigeria. Infect Dis Poverty. 2022;11(1):111. https://doi.org/10.1186/s40249-022-01034-3
6. WHO. Ending the neglect to attain the Sustainable Development Goals: A road map for neglected tropical diseases 2021-2030. Available from https://www.who.int/publications/i/item/9789240010352
7. Nduka F, Nebe OJ, Njepuome N, Dakul DA, Anagbogu IA, Ngege E, *et al.* Epidemiological mapping of schistosomiasis and soil-transmitted helminthiasis for intervention strategies in Nigeria. Nig J Parasitol. 2019;40(2):218. https://doi.org/10.4314/njpar.v40i2
8. Akwa Ibom State Ministry of Health (AKSMoH) (2023). Reports on Schistosomiasis in Akwa Ibom State. Unpublished.
9. WHO. Assessing schistosomiasis and soil-transmitted helminthiases control programmes: monitoring and evaluation framework. Available from https://www.who.int/publications/b/73248
10. WHO. Practical Precision Assessments for Schistosomiais: A manual for impact assessments. Available from https://espen.afro.who.int/sites/default/files/content/document/Practical%20and%20Precision%20Assessment%20Manual_0.pdf
11. Montresor A, Deol A, à Porta N, Lethanh N, Jankovic D. Markov Model Predicts Changes in STH Prevalence during Control Activities Even with a Reduced Amount of Baseline Information. PLOS Negl Trop Dis. 2016;10(4):e0004371. https://doi.org/10.1371/journal.pntd.0004371
12. WHO. Helminth control in school-age children: a guide for managers of control programmes. Available from https://www.who.int/publications/i/item/9789241548267
13. Harris-Roxas B, Viliani F, Bond A, Cave B, Divali M, Furu P. Health impact assessment: The state of the art. Impact Assess Proj Appraisal. 2012;30:43-52. https://doi.org/10.1080/14615517.2012.666035
14. Binshak N, Danaat DA, Tali DM, Helen DN. Impact Assessment of Schistosomiasis and Soil-Transmitted Helminthiasis Following Ten (10) Years (2009-2019) of Continuous Treatment with Praziquantel and Albendazole in Endemic Area, Plateau State. OALibJ. 2023;10:e9669. https://doi.org/10.4236/oalib.1109669
15. WHO. Water sanitation and hygiene for accelerating and sustaining progress on neglected tropical diseases. A global strategy 2015–2020. Available from https://www.who.int/publications/i/item/WHO-FWC-WSH-15.12

16. Bartlett AW, Mendes EP, Dahmash L, Palmeirim MS, de Almeida MC, Peliganga LB, *et al*. School-based preventive chemotherapy program for schistosomiasis and soil-transmitted helminth control in Angola: 6-year impact assessment. PLoS Negl Trop Dis. 2023;17(5):e0010849. https://doi.org/10.1371/journal.pntd.0010849 PMID: 37196040
17. Okoyo C, Campbell SJ, Williams K, Simiyu E, Owaga C, Mwandawiro C. Prevalence, intensity and associated risk factors of soil-transmitted helminth and schistosome infections in Kenya: Impact assessment after five rounds of mass drug administration in Kenya. PLoS Negl Trop Dis. 2020;14(10):e0008604. https://doi.org/10.1371/journal.pntd.0008604 PMID: 33027264
18. Mwandawiro C, Okoyo C, Kihara J, Simiyu E, Kepha S, Campbell SJ, *et al*. Results of a national school-based deworming programme on soil-transmitted helminths infections and schistosomiasis in Kenya: 2012-2017. Parasit Vectors. 2019;12(1):76. https://doi.org/10.1186/s13071-019-3322-1 PMID: 30732642
19. Olamiju F, Nebe OJ, Mogaji H, Marcus A, Amodu-Agbi P, Urude RO, *et al*. Schistosomiasis outbreak during COVID-19 pandemic in Takum, Northeast Nigeria: Analysis of infection status and associated risk factors. PLoS One. 2022;17(1):e0262524. https://doi.org/10.1371/journal.pone.0262524 PMID: 35061823
20. Jacob SM, Diehl J-C, Vdovine G, Agbana T, Popoola S, Jujjavaragu S, *et al.* Schistosomiasis and Soil-Transmitted Helminthiasis Among School Age Children: Impact of 3-5 Annual Rounds of Mass Drug Administration in Ekiti State, Southwest Nigeria. Trop Med Infect Dis. 2025;10(4):85. https://doi.org/10.3390/tropicalmed10040085
21. Makau-Barasa L, Assefa L, Aderogba M, Bell D, Solomon J, Urude RO, *et al.* Performance evaluation of the AiDx multi-diagnostic automated microscope for the detection of schistosomiasis in Abuja, Nigeria. Sci Rep. 2023;13(1):14833. https://doi.org/10.1038/s41598-023-42049-6
22. Onasanya A, Bengtson M, Agbana T, Oladunni O, van Engelen J, Oladepo O, *et al.* Towards Inclusive Diagnostics for Neglected Tropical Diseases: User Experience of a New Digital Diagnostic Device in Low-Income Settings. Trop Med Infect Dis. 2023;8(3):176. https://doi.org/10.3390/tropicalmed8030176
