# Supplementary material for: Impact assessment of effective rounds of mass drug administration against schistosomiasis and soil transmitted helminthiasis among school age children in Akwa Ibom State, Nigeria
Source: PLoS Negl Trop Dis. 2026 Aug 7;20(8):e0014603. doi: 10.1371/journal.pntd.0014603 (PMC13450779; doi:10.1371/journal.pntd.0014603)
Supplement: Supplementary file 3 [file pntd.0014603.s005.docx]

**SUBMISSION ID:**  PNTD-D-26-00512

Impact Assessment of Effective Rounds of Mass Drug Administration against Schistosomiasis and Soil Transmitted Helminthiasis among School Age Children in Akwa Ibom State, Nigeria.

We express our sincere gratitude to the editors and reviewers for their constructive feedback and insightful suggestions regarding our manuscript.

We have carefully addressed each point raised by the reviewers to enhance the technical quality, clarity and public health significance of our study.

**JOURNAL REQUIREMENTS:**

1) Please ensure that the CRediT author contributions listed for every co-author are completed accurately and in full.
   At this stage, the following Authors/Authors require contributions: Kenneth Nnamdi Opara, Clement Ameh Yaro, Nnamso Williams Ikpeida, Aliyu Mohammed, Hadiza Joy Marcus, Kingsley Azu, Salisu Ahmad, Ellie Leaning, Moses Aderogba, Louise-Makau Barasa, Antia Etop, Imeh Sylvester Udoh, Fatai Oyediran, Imabong O. Umah, Solomon M. Jacob, Gideon U. Ntuen, Veronica S. Augustine, and Halima Toro. Please ensure that the full contributions of each author are acknowledged in the "Add/Edit/Remove Authors" section of our submission form.
   The list of CRediT author contributions may be found here: <https://journals.plos.org/plosntds/s/authorship#loc-author-contributions>

**Done**

2) Please upload all main figures as separate Figure files in .tif or .eps format. For more information about how to convert and format your figure files please see our guidelines:
   <https://journals.plos.org/plosntds/s/figures>

**Done**

3) In the online submission form, you indicated that "The data sets in this study are available from the corresponding author on reasonable request.". All PLOS journals now require all data underlying the findings described in their manuscript to be freely available to other researchers, either
   1. In a public repository
   2. Within the manuscript itself
   3. Uploaded as supplementary information.
   This policy applies to all data except where public deposition would breach compliance with the protocol approved by your research ethics board. If your data cannot be made publicly available for ethical or legal reasons (e.g., public availability would compromise patient privacy), please explain your reasons by return email and your exemption request will be escalated to the editor for approval. Your exemption request will be handled independently and will not hold up the peer review process, but will need to be resolved should your manuscript be accepted for publication. One of the Editorial team will then be in touch if there are any issues.

**Supplementary file**

4) Figures 1, 2, 3, 4, and 5: please (a) provide a direct link to the base layer of the map (i.e., the country or region border shape) and ensure this is also included in the figure legend; and (b) provide a link to the terms of use / license information for the base layer image or shapefile. We cannot publish proprietary or copyrighted maps (e.g. Google Maps, Mapquest) and the terms of use for your map base layer must be compatible with our CC BY 4.0 license.
   Note: if you created the map in a software program like R or ArcGIS, please locate and indicate the source of the basemap shapefile onto which data has been plotted.

   If your map was obtained from a copyrighted source please amend the figure so that the base map used is from an openly available source. Alternatively, please provide explicit written permission from the copyright holder granting you the right to publish the material under our CC BY 4.0 license.

   If you are unsure whether you can use a map or not, please do reach out and we will be able to help you. The following websites are good examples of where you can source open access or public domain maps:
   * U.S. Geological Survey (USGS) - All maps are in the public domain. ([http://www.usgs.gov](http://www.usgs.gov/))
   * PlaniGlobe - All maps are published under a Creative Commons license so please cite “PlaniGlobe, [http://www.planiglobe.com](http://www.planiglobe.com/), CC BY 2.0” in the image credit after the caption. (<http://www.planiglobe.com/?lang=enl>)
   * Natural Earth - All maps are public domain. (<http://www.naturalearthdata.com/about/terms-of-use/>)

**Done**

**POINT-BY-POINT RESPONSE TO REVIEWERS**

| **Comments** | **Responses** |
| --- | --- |
| **REVIEWER 1**  **General comments** |  |
| • Line 114-116: Can authors provide range for species prevalence as they have done for overall STH | **See line 115 to 117**  Species-specific prevalence was 15.38% (95% CI: 14.52–16.28, range: 3.3% to 38.3%) for hookworm, 15.10% (95% CI: 14.24–15.99, range: 1.6% to 67.0%) for *Ascaris lumbricoides*, and 1.52% (95% CI: 1.25–1.85, range: 0.4% to 9.3%) for *Trichuris trichiura*. |
| Line 124: OR is missing for availability of school sanitation | **See line 123 to 129**  Compared with baseline data, STH prevalence declined by 30.15%. Significant risk factors associated with STH include; males (aOR = 1.196, 95% CI: 1.072 – 1.335), age group of 11 to 14 years (aOR = 1.391, 95% C.I.: 1.244 – 1.554), dry pit latrine (aOR = 1.464, 95% C.I. = 1.239 – 1.731), schools where children urinate (aOR = 2.620, 95% C.I.: 2.362 – 2.929) and defecate (aOR = 1.238, 95% C.I. = 1.097 – 1.396) around school or wait to defecate after school (aOR = 1.830, 95% C.I. = 1.365 – 2.454), schoolchildren that drinks from surface water (aOR = 1.731, 95% C.I. = 1.486 – 2.017) or boreholes that uses hand pump (aOR = 1.666, 95% C.I. = 1.412 – 1.965). |
| Line 154: Revise to; In terms of burden… | **See line 157**  In terms of burden, Nigeria has been at the forefront of the African region, with 134 million people at risk of SCH and widespread STH transmission [5] |
| Line 156: SAC not defined before now | **See line 160**  The WHO NTD Roadmap 2021–2030 targets both diseases for elimination as a public health problem (EPHP) for SCH (<1% prevalence in School-Age Children (SAC)) and STH (<2% moderate/heavy intensity) by 2030, with impact assessments to be done after 3–5 MDA rounds [6]. |
| Line 156-58: abrupt jump into impact assessment, without description of the intervention and implementation strategy. | **See line 162 to 169**  Prior to the impact assessment, the preventive chemotherapy (PC) strategy implemented in Akwa Ibom State was led by the Federal Ministry of Health (FMoH) in collaboration with the State Ministry of Health and supported by Helen Keller Intl focused on large scale Mass Administration of Medicines (MAM) targeting school-aged children (SAC) aged 5 to 14 years. For STH, the strategy mandated biannual PC in areas with prevalence above 50%, and annual PC in regions with a 20% to 49.9% prevalence with over 75% coverage across implementation units (IUs) using albendazole or menbendazole, while SCH control among SAC relied on a case-management approach utilizing praziquantel [7]. |
| Line 160-161: appears confusing and disjointed. | **See line 169 to 177**  Earlier baseline studies in Akwa Ibom State covered all the 31 LGAs. The baseline surveys (2009-2017) in Akwa Ibom State revealed a high level of STH endemicity (25.1 - 91.4%), and a low level of SCH prevalence (0 - 1.2%) [8]. Also, between 2014 and 2023, a survey for SCH endemicity reflected low endemicity [9]. In 2024, an impact assessment survey was carried out in five (5) LGAs that has achieved five effective rounds of mass drug administration (MDA). The current assessment targets the remaining 26 LGAs that have similarly completed five effective treatment rounds, with the aim of evaluating the impact of the intervention and informing decisions on the next phase of the elimination programme. |
| Line 162, authors should not compare STH endemicity to SCH. Kindly replace that word with “and” | **See line 169 to 172**  Earlier baseline studies in Akwa Ibom State covered all the 31 LGAs. The baseline surveys (2009-2017) in Akwa Ibom State revealed a high level of STH endemicity (25.1 - 91.4%), and a low level of SCH prevalence (0 - 1.2%) [8]. |
| Why are the authors focusing on 26 out of 31 | **See line 173 to 177**  In 2024, an impact assessment survey was carried out in five (5) LGAs that has achieved five effective rounds of mass drug administration (MDA). The current assessment targets the remaining 26 LGAs that have similarly completed five effective treatment rounds, with the aim of evaluating the impact of the intervention and informing decisions on the next phase of the elimination programme. |
| LINE 71-73: It is unclear if PC were implemented for SCH. | **Dear reviewer, it is now clearly stated. See line 162 to 169**  Prior to the impact assessment, the preventive chemotherapy (PC) strategy implemented in Akwa Ibom State was led by the Federal Ministry of Health (FMoH) in collaboration with the State Ministry of Health and supported by Helen Keller Intl focused on large scale Mass Administration of Medicines (MAM) targeting school-aged children (SAC) aged 5 to 14 years. For STH, the strategy mandated biannual PC in areas with prevalence above 50%, and annual PC in regions with a 20% to 49.9% prevalence with over 75% coverage across implementation units (IUs) using albendazole or menbendazole, while SCH control among SAC relied on a case-management approach utilizing praziquantel [7]. |
| The LGAs between lines 168-173 should be removed, the authors have repeated them between lines 187-190 | Removed |
| Line 174-177: also needs significant revision. Authors should provide more solid rationales for conducting impact assessment and cite appropriate references. It is also important to state that Impact assessment following 5 effective rounds of PC. | **See line 178 to 182**  This study evaluates the impact of PC on the prevalence, intensity and endemicity of SCH and STH across 26 LGAs of Akwa Ibom State. The assessment follows five effective rounds of PC and is designed to determine whether the elimination as a public health problem (EPHP) targets have been achieved in accordance with the WHO monitoring and evaluation framework [7]. The survey is part of the national and state NTD strategic plan to provide data needed for planning, implementation, and monitoring the progress of the SCH and STH control programme in Akwa Ibom State. |
| Please provide ethical approval number, line 193 | **See line 198 to 202**  The Akwa Ibom State Research Ethics Committee provided ethical approval (AKHREC/08/09/25/376). Community gatekeepers were provided with administrative clearance and letters of introduction. Parents or guardians of participating school-aged children (SAC) provided written informed consent and assent of children was obtained where necessary. It was voluntary and confidential. |
| For reproducibility, authors should be more explicit about their methods. This is particularly important for other settings that may wish to adopt strategies that had worked elsewhere. Kindly revise line 203-206; | **See line 210 to 215**  A cross-sectional impact assessment among school-aged children was undertaken in October 2025 after the implementation of 5-8 effective rounds of mass drug administration (MDA). The study employed a two-stage sampling design aligned with the WHO [7] guidelines for impact assessment. Initially, five (5) wards were purposively selected from each Local Government Area (LGA), taking into account their epidemiological importance and baseline endemicity profiles identified during the baseline survey. |
| Line 208-210: How as the purposive sampling done; see comments above. For STH, decisions on impact assessment are made at the LGA level, however, for SCH, recent recommendations suggest sampling 15 PSUs per LGA. It is unclear what the authors did to arrive at the number of PSUs. Authors need to also clearly state the methodology they employed while sampling. | **See line 217 to 221**  In each implementation unit (IU), the school with the highest prevalence of STH and SCH was purposively selected as the primary sampling unit (PSU). The selection was made from the list of eligible schools within the IU resulting in a total of five schools per LGA and 130 PSUs in 26 LGAs. |
| Fig 1 : can you change the brown colour used for the map, too dark that it shadows the label of the LGAs | **See line 229 to 230**  The colours on the map has been changed to brighter colours (lemon and orange). |
| Line 240: whih application was deployed for data collection | **See line 261 to 262**  The electronic data collection was done using the KoboCollect App on Android based devices linked to a cloud server. |
| The data analysis description here was for STH. It seems authors missed out SCH. Similarly, since the LGAs have met the 5 rounds of effective pc, it is erroneous to compare their endemicity thresholds at the level the authors have done, the comparisons should be done at (1) <2%, (2) 2% to <10%, (3) 10% to <20%, and (4) ≥20%. | **See line 270 to 271**  Cleaning of data was done in Microsoft Excel. The prevalence between the categories of infection i.e. based on sex, age groups, and LGAs were determined through descriptive statistics. WHO thresholds were used to classify endemicity; STH(< 2%, ≥ 2% to < 10%, ≥ 10 % to < 20%, and ≥ 20%) and SCH (<10%, ≥ 10% to 49.9, and ≥ 50%) (WHO, 2024). The severity of the infections was determined based on WHO guidelines [7]. |
| Please see below excerpts that should guide the analysis  The analysis and interpretation of data for preventive chemotherapy (PC) adjustments followed the most recent World Health Organization (WHO) guidelines for STH prevalence impact assessment surveys [21]. Local Government Areas (LGAs) were categorized into four prevalence groups: (1) <2%, (2) 2% to <10%, (3) 10% to <20%, and (4) ≥20%. For LGAs in the first group, PC is recommended only during specific events, alongside the establishment and maintenance of surveillance systems. For LGAs in the second group, biennial PC over 5 years is recommended, with continuous surveillance. LGAs in the third group are advised to implement annual PC for 5 years with ongoing surveillance, while those in the fourth group are required to administer annual PC for 5 years. The aggregated proportion of moderate and heavy infections (MHI) was also calculated for each LGA, using a threshold of ≤2%, where the upper bound of the 95% confidence interval (CI) was below this limit.  Ref: World Health Organization (WHO). Assessing schistosomiasis and soil-transmitted helminthiases control programmes: monitoring and evaluation framework. Geneva: World Health Organization; 2024 [cited 2024 Nov 11. ]. Licence: CC BY-NC-SA 3.0 IGO. Available from: <https://iris.who.int/bitstream/handle/10665/379352/9789240099364-eng.pdf>  infact the first reference on your list; exemplifies how we did the interpretation recently. <https://journals.plos.org/plosntds/article?id=10.1371/journal.pntd.0012533#references> | **Correction effected as suggested. See line 267 to 277**  Cleaning of data was done in Microsoft Excel. The prevalence between the categories of infection i.e. based on sex, age groups, and LGAs were determined through descriptive statistics. WHO thresholds were used to classify endemicity; STH(< 2%, ≥ 2% to < 10%, ≥ 10 % to < 20%, and ≥ 20%) and SCH (<10%, ≥ 10% to 49.9, and ≥ 50%) (WHO, 2024). The severity of the infections was determined based on WHO guidelines [7]. The 95% confidence intervals (CI) were used to calculate prevalence estimates. Chi-square (χ^2^) analysis was performed to test associations. Bivariate and multivariate logistic regression was used to estimate odds ratios (ORs) and adjusted odds ratios (aORs) with 95% CI. The significant level was established at p < 0.05. Statistical analysis was conducted using Statistical Package for Social Sciences (SPSS) version 27.  **Also see line 373 to 386**  The study also examined the socio-demographic characteristics and WASH factors in relation to the infection status. The risk of acquiring STH infections was significantly associated with sex, age group of school children, type of toilet facility, when and where children urinate and defecate in schools (Table 8). Male school children had a higher likelihood of being infected with STH (aOR = 1.196, 95% CI: 1.072 – 1.335). Children in the age group of 11 to 14 years (aOR = 1.391, 95% C.I.: 1.244 – 1.554) are at higher risk of being infected with STH. School children that uses dry pit (aOR = 1.464, 95% C.I. = 1.239 – 1.731), urinate around school (aOR = 2.620, 95% C.I.: 2.362 – 2.929), defecate around school (aOR = 1.238, 95% C.I. = 1.097 – 1.396) or wait to defecate after school (aOR = 1.830, 95% C.I. = 1.365 – 2.454) had higher likelihood of being infected with STH. Also, school children that drink water from borehole with hand pump (aOR = 1.666, 95% C.I. = 1.412 – 1.965) and those that drink from surface water (aOR = 1.731, 95% C.I. = 1.486 – 2.017) had higher likelihood of being infected with STH (Table 8).  **Programmatic interpretation of our findings has now been included. See line 400 to 415**   3.1. **Programmatic Interpretation of Results**   The results of this impact assessment has shown significant decrease in the STH prevalence thresholds across the 26 LGAs of Akwa Ibom State. Out of 17 LGAs in the threshold of ≥50% at baseline, 11 LGAs and 3 LGAs dropped to the threshold of 20 to 49.9% and 0 – 19.9% respectively. Out of the 9 LGAs in the prevalence threshold of 20 – 49.9% at baseline, 8 LGAs dropped to the prevalence threshold of 0 to 19.9% (Table 9). Based on this report, 11 LGAs requires no PC, 12 LGAs requires PC once per annum for 5 years while 3 LGAs requires PC twice per annum for a period of 5 years (Table 9, Figure 7). |
| Since the data analysis is faulty, so does the interpretation of findings, hence it would be impossible to proceed with the review, | Analysis was improved upon. For example multivariate logistic regression was calculated for the risk factors. Also, interpretation of impact assessment was improved on as presented in table 8. |
| Also authors should kindly perform adjusted OR, to help control cofounders. | aOR calculated as suggested. See Table 8 |
|  |  |
| **REVIEWER 2** |  |
| **Methods**  The authors did not clearly articulate the objectives concerning the issues under investigation, particularly in relation to other critical elements that influence program effectiveness and the testable hypotheses. While the study design is generally appropriate, it is noteworthy that only one objective was specified in the manuscript. The target population, referred to as SAC, was adequately described, and the sample size was sufficient. Furthermore, appropriate statistical analyses were utilized to substantiate the findings. However, although ethical approval was obtained, the manuscript did not include the approval number. | **Objectives now clearly stated, ethical approval number provided. The programme effectiveness and factors influencing it now outlined. See line 178 to 184**  This study evaluates the impact of PC on the prevalence, intensity and endemicity of SCH and STH across 26 LGAs of Akwa Ibom State. The assessment follows five effective rounds of PC and is designed to determine whether the elimination as a public health problem (EPHP) targets have been achieved in accordance with the WHO monitoring and evaluation framework [7]. The survey is part of the national and state NTD strategic plan to provide data needed for planning, implementation, and monitoring the progress of the SCH and STH control programme in Akwa Ibom State.  **The ethical approval number is now provided. See line 197 to 199**  The Akwa Ibom State Research Ethics Committee provided ethical approval (AKHREC/08/09/25/376). Community gatekeepers were provided with administrative clearance and letters of introduction. Parents or guardians of participating school-aged children (SAC) provided written informed consent and assent of children was obtained where necessary. It was voluntary and confidential. |
| **Results**  The analysis was conducted in accordance with the established analysis plan. The results were presented clearly within the manuscript, utilizing well-structured tables and figures to enhance understanding. | Thank you for taking your time to review this manuscript. |
| **Conclusion**  The conclusions of the study were well-supported by the presented data. However, the authors did not articulate the limitations associated with their research. Although they engaged in a discussion of their findings, they failed to provide a sufficient comparative analysis regarding how their results correspond with or differ from other related studies conducted in Nigeria and throughout Africa.  Furthermore, the authors did not adequately highlight the public health implications of their findings. It remains unclear whether Akwa Ibom State is making meaningful progress toward the elimination of soil-transmitted helminths (STH) by the year 2030. If progress is not evident, it is imperative that the authors address the limitations and barriers that may be impeding advancement in this area | **The conclusion is now presented to reflect the public health implications and limitations of the survey. See line 477 to 487**  Preventive chemotherapy (PC) program for schistosomiasis and soil-transmitted helminthiasis (STH) in Akwa Ibom state has proven highly effective for schistosomiasis with 0.0% prevalence compared to baseline of 0.0 to 1.2% but partly effective for soil-transmitted helminthiasis with overall prevalence of 27.62% (ranges from 4.92 – 71.65%) compared to baseline of 25.1 to 91.4% with a 30.15% reduction in prevalence. The survey documented a reduction in STH prevalence across all 26 LGAs; 12 LGAs now require annual PC, 3 require biannual PC, and 11 require no PC. For SCH, continued case management is recommended across all 26 LGAs. The widespread absence of functional WASH facilities in schools remains a critical risk factor threatening the long-term sustainability of MDA-driven gains. These findings underscore the imperative for sustained treatment and control programmes, underpinned by substantive improvements in water, sanitation, and hygiene infrastructure.  **In our discussion, we made comparison of our study to previous studies in other parts of the country.** |
| **General comments**  The manuscript demonstrates strong writing; however, to enhance its impact, the study's objectives must be explicitly defined. The discussion section needs to be more effectively structured, ensuring that comparisons with existing research are clear and compelling. It is also essential to emphasize any new knowledge or innovations that the study contributes | **Done. The objectives clearly outlined. See line 178 to 184**  This study evaluates the impact of PC on the prevalence, intensity and endemicity of SCH and STH across 26 LGAs of Akwa Ibom State. The assessment follows five effective rounds of PC and is designed to determine whether the elimination as a public health problem (EPHP) targets have been achieved in accordance with the WHO monitoring and evaluation framework [7]. The survey is part of the national and state NTD strategic plan to provide data needed for planning, implementation, and monitoring the progress of the SCH and STH control programme in Akwa Ibom State.  **Comparison with existing research was previously done. We have added more as suggested. Kindly see discussion section. See line 418 to 474.** |
|  |  |
