# Supplementary material for: Impact assessment of effective rounds of mass drug administration against schistosomiasis and soil transmitted helminthiasis among school age children in Akwa Ibom State, Nigeria
Source: PLoS Negl Trop Dis. 2026 Aug 7;20(8):e0014603. doi: 10.1371/journal.pntd.0014603 (PMC13450779; doi:10.1371/journal.pntd.0014603)
Supplement: Supplementary file 4 [file pntd.0014603.s006.docx]

**Submission ID:**  PNTD-D-26-00512R1

**Manuscript Title**: Impact Assessment of Effective Rounds of Mass Drug Administration against Schistosomiasis and Soil Transmitted Helminthiasis among School Age Children in Akwa Ibom State, Nigeria

PLOS Neglected Tropical Diseases

**Journal Requirements:**

If the reviewer comments include a recommendation to cite specific previously published works, please review and evaluate these publications to determine whether they are relevant and should be cited. There is no requirement to cite these works unless the editor has indicated otherwise.

1) Please ensure that the CRediT author contributions listed for every co-author are completed accurately and in full.

At this stage, the following Authors/Authors require contributions: Kenneth Nnamdi Opara, Clement Ameh Yaro, Nnamso Williams Ikpeida, Aliyu Mohammed, Hadiza Joy Marcus, Kingsley Azu, Salisu Ahmad, Ellie Leaning, Moses Aderogba, Louise-Makau Barasa, Antia Etop, Imeh Sylvester Udoh, Fatai Oyediran, Imabong O. Umah, Solomon M. Jacob, Gideon U. Ntuen, Veronica S. Augustine, and Halima Toro. Please ensure that the full contributions of each author are acknowledged in the "Add/Edit/Remove Authors" section of our submission form.

The list of CRediT author contributions may be found here: <https://journals.plos.org/plosntds/s/authorship#loc-author-contributions>

**Done**

2) Please upload all main figures as separate Figure files in .tif or .eps format. For more information about how to convert and format your figure files please see our guidelines:
   <https://journals.plos.org/plosntds/s/figures>

**Done**

3) We have noticed that you have uploaded Supporting Information files, but you have not included a list of legends. Please add a full list of legends for your Supporting Information files after the references list.

**There is no abbreviation requiring legends. Therefore it was not provided.**

**POINT-BY-POINT RESPONSE TO REVIEWERS**

| **Comments** | **Responses** |
| --- | --- |
| **REVIEWER 1**  **General comments** |  |
| - The authors have satisfactorily addressed most of the comments but the most important one regarding the programmatic interpretation of the data is not revised. They have interpreted their data based on old thresholds used before initiating MDA i.e., 0-19.9; 20-49.9 and >50. However, the WHO thresholds for LGA that have had effective MDA for more than 5 years is < 2%, ≥ 2% to < 10%, ≥ 10 % to < 20%, and ≥ 20%. So most of the tables and figures presented needs to be revised accordingly. | Figure 2, 3, 4, 5 and 7 corrected to reflect the new WHO thresholds for LGA.  Also, Table 9 was corrected appropriately to suit the new WHO thresholds for LGA.  **Line 119 to 121**  **Line 416 to 429**  **Programmatic Interpretation of Results**  The results of this impact assessment has shown significant decrease in the STH prevalence thresholds across the 26 LGAs of Akwa Ibom State. Out of 17 LGAs in the threshold of ≥50% at baseline, 14 remained in the threshold of ≥20% while 3 LGAs moved down to the threshold of ≥10 to <20%. Also, 9 LGAs in the threshold of 20 to 49.9% at baseline, 1 LGA moved down to the threshold of ≥2 to <10%, 7 LGAs moved down to the threshold of ≥10 to <20% while 1 LGA remained in the threshold of ≥20%. Based on this report, 1 LGA requires MDA once in 2 years for a period of 5 years with sustained surveillance activities, 10 LGAs requires MDA once yearly for a period of 5 years with sustained surveillance while 15 LGAs requires MDA twice yearly for a period of 5 years with sustained surveillance activities (Table 9, Figure 7).  **Line 454 to 458**  **Line 507 to 509** |
| - Also, for the intensity. It is unclear how authors interpreted the intensity presented on table 6, each of the species has different egg count for intensity threshold. Hence, authors are expected to present intensity per STH species based on that egg count. Please see; the intensity of *Ascaris lumbricoides* was classified as low (1–4,999 EPG), moderate (5,000–49,999 EPG), or high (≥50,000 EPG). For hookworms, intensities were classified as low (1–1,999 EPG), moderate (2,000–3,999 EPG), or high (≥4,000 EPG) *Trichuris trichiura* infections were categorized as low (1–999 EPG), moderate (1,000–9,999 EPG), or high (≥10,000 EPG) [15].   Please see a copy of a similar manuscript published; <https://www.mdpi.com/2414-6366/11/1/24> | Calculations on intensity now done according to species of STHs. As shown below. Also see table 6 for details.  **Line 370 to 376 and Table 6**.   3.1. **STH Infection Load Estimation**   A total of 98.66% (958 school children) and 1.44% (13 school children) had light and moderate infection of *A. lumbricoides* respectively, had light infection, while 1.44% (14 individuals) had moderate infection. Also, 99.09% (980 school children) and 0.91% (9 school children) had light and moderate infections of hookworms. All 98 school children infected with *T. trichiura* had light infection. High intensity infection was not recorded across the 26 LGAs (Table 6). |
| **REVIEWER 2**  **General comments** |  |
| - The manuscript is well written, with robust statistical analysis. However the authors should address few comments and observations made on the manuscript. | Comments addressed as shown below |
| - The claim regarding the potential elimination of schistosomiasis in Akwa Ibom State is fundamentally misleading; the state is not endemic for schistosomiasis based on baseline findings. Moreover, any assertion of elimination must be substantiated through a comprehensive evaluation that adheres to the criteria and assessment procedures established by the World Health Organization (WHO). This evaluation must include thorough assessments of humans, snails, One Health initiatives, and Water, Sanitation, and Hygiene (WASH) interventions. Credible data must be generated and submitted to the WHO, along with preparations for a dossier submission for validation and certification, before making definitive claims about elimination. | Now corrected to read (**Line 140**):  In this current study, no cases of schistosomiasis were detected in school children in 26 LGAs. |
| - **Line 96-97**: For how long was PC intervention implemented in the State? | Dear Reviewer,  Five effective rounds of PC was implemented in the state. It is stated in the last sentence of the background, under abstract as shown below (**Line 168 to 171**):  **Abstract**  **Background:** Schistosomiasis (SCH) and Soil-transmitted helminthiasis (STH) remain major neglected tropical diseases across sub-Saharan Africa. In Akwa Ibom State, preventive chemotherapy (PC) has been implemented in endemic Local Government Areas (LGAs). This study assessed the impact of five effective rounds of PC on the prevalence and intensity of SCH and STH in 26 endemic LGAs. |
| - **Line 139**: This a very strong statement, equally misleading, which the available evidence does not justify elimination of schistosomiasis in Akwa Ibom State. - Have the authors conducted due diligence investigations on snail intermediate host of schistosomiasis, animals and simultaneously with WASH interventions according to the WHO elimination criteria and documented evidence presented to the WHO for verification and validation? | Now corrected to read (**Line 140**):  In this current study, no cases of schistosomiasis were detected in school children in 26 LGAs. |
| - **Line 168**: Are the authors referring to the National baseline survey for Nigeria, because Akwa Ibom State baseline assessment did not stretch between 2009-2017? Kindly clarify this statement | Dear Reviewer,  The authors are referring to the National Baseline Survey conducted between 2013-2015 (**Line 168 to 171**).  It is now corrected to read as shown below:  Earlier baseline studies in Akwa Ibom State covered all the 31 LGAs. The baseline surveys (2013-2015) in Akwa Ibom State revealed a high level of STH endemicity (25.1 - 91.4%), and a low level of SCH prevalence (0.0 - 1.2%) [8, 9]. |
| - **Line 415-419**: The authors should cross-check the no of LGAs being considered not requiring PC. The actually number is 9 not 11 as reported. Ibiono Ibom which recorded 19.91% should not be overlooked or under classified but approximated to 20%. | Dear Reviewer,  The authors followed the WHO thresholds for LGA: < 2% - no PC, ≥ 2% to < 10% - once in 2 years PC, ≥ 10 % to < 20% - once yearly PC, and ≥ 20% - twice yearly PC. |

**Figure resubmission:**

While revising your submission, we strongly recommend that you use PLOS’s NAAS tool (https://ngplosjournals.pagemajik.ai/artanalysis) to test your figure files. NAAS can convert your figure files to the TIFF file type and meet basic requirements (such as print size, resolution), or provide you with a report on issues that do not meet our requirements and that NAAS cannot fix.

After uploading your figures to PLOS’s NAAS tool - https://ngplosjournals.pagemajik.ai/artanalysis, NAAS will process the files provided and display the results in the "Uploaded Files" section of the page as the processing is complete. If the uploaded figures meet our requirements (or NAAS is able to fix the files to meet our requirements), the figure will be marked as "fixed" above. If NAAS is unable to fix the files, a red "failed" label will appear above. When NAAS has confirmed that the figure files meet our requirements, please download the file via the download option, and include these NAAS processed figure files when submitting your revised manuscript.

**Done**

**Reproducibility:**

To enhance the reproducibility of your results, we recommend that authors of applicable studies deposit laboratory protocols in protocols.io, where a protocol can be assigned its own identifier (DOI) such that it can be cited independently in the future. Additionally, PLOS ONE offers an option to publish peer-reviewed clinical study protocols. Read more information on sharing protocols at https://plos.org/protocols?utm_medium=editorial-email&utm_source=authorletters&utm_campaign=protocols
