# Supplementary material for: Impact assessment of effective rounds of mass drug administration against schistosomiasis and soil transmitted helminthiasis among school age children in Akwa Ibom State, Nigeria
Source: PLoS Negl Trop Dis. 2026 Aug 7;20(8):e0014603. doi: 10.1371/journal.pntd.0014603 (PMC13450779; doi:10.1371/journal.pntd.0014603)
Supplement: Peer review history

# Peer review: Decision Letter 0

**DOI:** 10.1371/journal.pntd.0014603.r001

**Authors:** Uwem Ekpo, Francesca Tamarozzi

15 Apr 2026

PNTD-D-26-00512

Impact Assessment of Effective Rounds of Mass Drug Administration against Schistosomiasis and Soil Transmitted Helminthiasis among School Age Children in Akwa Ibom State, Nigeria

PLOS Neglected Tropical Diseases

Dear Dr. Yaro,

Thank you for submitting your manuscript to PLOS Neglected Tropical Diseases. After careful consideration, we feel that it has merit but does not fully meet PLOS Neglected Tropical Diseases's publication criteria as it currently stands. Therefore, we invite you to submit a revised version of the manuscript that addresses the points raised during the review process.

Please submit your revised manuscript within by Jun 14 2026 11:59PM. If you will need more time than this to complete your revisions, please reply to this message or contact the journal office at plosntds@plos.org. When you're ready to submit your revision, log on to https://www.editorialmanager.com/pntd/ and select the 'Submissions Needing Revision' folder to locate your manuscript file.

Please include the following items when submitting your revised manuscript:

* A letter that responds to each point raised by the editor and reviewer(s). You should upload this letter as a separate file labeled 'Response to Reviewers'. This file does not need to include responses to any formatting updates and technical items listed in the 'Journal Requirements' section below.

* A marked-up copy of your manuscript that highlights changes made to the original version. You should upload this as a separate file labeled 'Revised Manuscript with Track Changes'.

* An unmarked version of your revised paper without tracked changes. You should upload this as a separate file labeled 'Manuscript'.

If you would like to make changes to your financial disclosure, competing interests statement, or data availability statement, please make these updates within the submission form at the time of resubmission. Guidelines for resubmitting your figure files are available below the reviewer comments at the end of this letter.

We look forward to receiving your revised manuscript.

Kind regards,

Uwem Friday Ekpo, PhD

Academic Editor

PLOS Neglected Tropical Diseases

Francesca Tamarozzi

Section Editor

PLOS Neglected Tropical Diseases

Shaden Kamhawi

co-Editor-in-Chief

PLOS Neglected Tropical Diseases

orcid.org/0000-0003-4304-636XX

Paul Brindley

co-Editor-in-Chief

PLOS Neglected Tropical Diseases

orcid.org/0000-0003-1765-0002

**Journal Requirements:**

1) Please ensure that the CRediT author contributions listed for every co-author are completed accurately and in full.

At this stage, the following Authors/Authors require contributions: Kenneth Nnamdi Opara, Clement Ameh Yaro, Nnamso Williams Ikpeida, Aliyu Mohammed, Hadiza Joy Marcus, Kingsley Azu, Salisu Ahmad, Ellie Leaning, Moses Aderogba, Louise-Makau Barasa, Antia Etop, Imeh Sylvester Udoh, Fatai Oyediran, Imabong O. Umah, Solomon M. Jacob, Gideon U. Ntuen, Veronica S. Augustine, and Halima Toro. Please ensure that the full contributions of each author are acknowledged in the "Add/Edit/Remove Authors" section of our submission form.

The list of CRediT author contributions may be found here: https://journals.plos.org/plosntds/s/authorship#loc-author-contributions

2) Please upload all main figures as separate Figure files in .tif or .eps format. For more information about how to convert and format your figure files please see our guidelines:

https://journals.plos.org/plosntds/s/figures

3) In the online submission form, you indicated that "The data sets in this study are available from the corresponding author on reasonable request.". All PLOS journals now require all data underlying the findings described in their manuscript to be freely available to other researchers, either

1. In a public repository

2. Within the manuscript itself

3. Uploaded as supplementary information.

This policy applies to all data except where public deposition would breach compliance with the protocol approved by your research ethics board. If your data cannot be made publicly available for ethical or legal reasons (e.g., public availability would compromise patient privacy), please explain your reasons by return email and your exemption request will be escalated to the editor for approval. Your exemption request will be handled independently and will not hold up the peer review process, but will need to be resolved should your manuscript be accepted for publication. One of the Editorial team will then be in touch if there are any issues.

4) Figures 1, 2, 3, 4, and 5: please (a) provide a direct link to the base layer of the map (i.e., the country or region border shape) and ensure this is also included in the figure legend; and (b) provide a link to the terms of use / license information for the base layer image or shapefile. We cannot publish proprietary or copyrighted maps (e.g. Google Maps, Mapquest) and the terms of use for your map base layer must be compatible with our CC BY 4.0 license.

Note: if you created the map in a software program like R or ArcGIS, please locate and indicate the source of the basemap shapefile onto which data has been plotted.

If your map was obtained from a copyrighted source please amend the figure so that the base map used is from an openly available source. Alternatively, please provide explicit written permission from the copyright holder granting you the right to publish the material under our CC BY 4.0 license.

If you are unsure whether you can use a map or not, please do reach out and we will be able to help you. The following websites are good examples of where you can source open access or public domain maps:

* U.S. Geological Survey (USGS) - All maps are in the public domain. (http://www.usgs.gov)

* PlaniGlobe - All maps are published under a Creative Commons license so please cite “PlaniGlobe, http://www.planiglobe.com, CC BY 2.0” in the image credit after the caption. (http://www.planiglobe.com/?lang=enl)

* Natural Earth - All maps are public domain. (http://www.naturalearthdata.com/about/terms-of-use/)

**Reviewers' Comments:**

Reviewer's Responses to Questions

**Key Review Criteria Required for Acceptance?**

As you describe the new analyses required for acceptance, please consider the following:

**Methods**

-Are the objectives of the study clearly articulated with a clear testable hypothesis stated?

-Is the study design appropriate to address the stated objectives?

-Is the population clearly described and appropriate for the hypothesis being tested?

-Is the sample size sufficient to ensure adequate power to address the hypothesis being tested?

-Were correct statistical analysis used to support conclusions?

-Are there concerns about ethical or regulatory requirements being met?

Reviewer #1: Yes, but needs improvement, i have made suggestions in my comments

Reviewer #2: The authors did not clearly articulate the objectives concerning the issues under investigation, particularly in relation to other critical elements that influence program effectiveness and the testable hypotheses. While the study design is generally appropriate, it is noteworthy that only one objective was specified in the manuscript. The target population, referred to as SAC, was adequately described, and the sample size was sufficient. Furthermore, appropriate statistical analyses were utilized to substantiate the findings. However, although ethical approval was obtained, the manuscript did not include the approval number.

**********

**Results**

-Does the analysis presented match the analysis plan?

-Are the results clearly and completely presented?

-Are the figures (Tables, Images) of sufficient quality for clarity?

Reviewer #1: Yes, but needs improvement, i have made suggestions in my comments

Reviewer #2: The analysis was conducted in accordance with the established analysis plan. The results were presented clearly within the manuscript, utilizing well-structured tables and figures to enhance understanding.

**********

**Conclusions**

-Are the conclusions supported by the data presented?

-Are the limitations of analysis clearly described?

-Do the authors discuss how these data can be helpful to advance our understanding of the topic under study?

-Is public health relevance addressed?

Reviewer #1: Yes, but needs improvement, i have made suggestions in my comments

Reviewer #2: The conclusions of the study were well-supported by the presented data. However, the authors did not articulate the limitations associated with their research. Although they engaged in a discussion of their findings, they failed to provide a sufficient comparative analysis regarding how their results correspond with or differ from other related studies conducted in Nigeria and throughout Africa.

Furthermore, the authors did not adequately highlight the public health implications of their findings. It remains unclear whether Akwa Ibom State is making meaningful progress toward the elimination of soil-transmitted helminths (STH) by the year 2030. If progress is not evident, it is imperative that the authors address the limitations and barriers that may be impeding advancement in this area.

**********

**Editorial and Data Presentation Modifications?**

Use this section for editorial suggestions as well as relatively minor modifications of existing data that would enhance clarity. If the only modifications needed are minor and/or editorial, you may wish to recommend “Minor Revision” or “Accept”.

Reviewer #1: (No Response)

Reviewer #2: Accept after minor revision

**********

**Summary and General Comments**

Use this section to provide overall comments, discuss strengths/weaknesses of the study, novelty, significance, general execution and scholarship. You may also include additional comments for the author, including concerns about dual publication, research ethics, or publication ethics. If requesting major revision, please articulate the new experiments that are needed.

Reviewer #1: • Line 114-116: Can authors provide range for species prevalence as they have done for overall STH

• Line 124: OR is missing for availability of school sanitation

• Line 154: Revise to; In terms of burden…

• Line 156: SAC not defined before now

• Line 156-58: abrupt jump into impact assessment, without description of the intervention and implementation strategy.

• Line 160-161: appears confusing and disjointed.

• Line 162, authors should not compare STH endemicity to SCH. Kindly replace that word with “and”

• why are the authors focusing on 26 out of 31

• LINE 71-73: It is unclear if PC were implemented for SCH.

• The LGAs between lines 168-173 should be removed, the authors have repeated them between lines 187-190

• Line 174-177: also needs significant revision. Authors should provide more solid rationales for conducting impact assessment and cite appropriate references. It is also important to state that Impact assessment following 5 effective rounds of PC.

• Please provide ethical approval number, line 193

• For reproducibility, authors should be more explicit about their methods. This is particularly important for other settings that may wish to adopt strategies that had worked elsewhere. Kindly revise line 203-206;

• Line 208-210: How as the purposive sampling done; see comments above. For STH, decisions on impact assessment are made at the LGA evel, however, for sch, rcent recommendations suggest sampling 15 PSUs per LGA. It is unclear what the authors did to arrive at the number of PSUs. Authors need to also clearly state the methodology they employed while sampling.

• Fig 1 : can you change the brown colour used for the map, too dark that it shadows the label of the LGAs

• Line 240: whih application was deployed for data collection

• The data analysis description here was for STH. It seems authors missed out SCH. Similarly, since the LGAs have met the 5 rounds of effective pc, it is erroneous to compare their endemicity thresholds at the level the authors have done, the comparisons should be done at (1) <2%, (2) 2% to <10%, (3) 10% to <20%, and (4) ≥20%.

Please see below excerpts that should guide the analysis

The analysis and interpretation of data for preventive chemotherapy (PC) adjustments followed the most recent World Health Organization (WHO) guidelines for STH prevalence impact assessment surveys [21]. Local Government Areas (LGAs) were categorized into four prevalence groups: (1) <2%, (2) 2% to <10%, (3) 10% to <20%, and (4) ≥20%. For LGAs in the first group, PC is recommended only during specific events, alongside the establishment and maintenance of surveillance systems. For LGAs in the second group, biennial PC over 5 years is recommended, with continuous surveillance. LGAs in the third group are advised to implement annual PC for 5 years with ongoing surveillance, while those in the fourth group are required to administer annual PC for 5 years. The aggregated proportion of moderate and heavy infections (MHI) was also calculated for each LGA, using a threshold of ≤2%, where the upper bound of the 95% confidence interval (CI) was below this limit.

Ref: World Health Organization (WHO). Assessing schistosomiasis and soil-transmitted helminthiases control programmes: monitoring and evaluation framework. Geneva: World Health Organization; 2024 [cited 2024 Nov 11. ]. Licence: CC BY-NC-SA 3.0 IGO. Available from: https://iris.who.int/bitstream/handle/10665/379352/9789240099364-eng.pdf

infact the first reference on your list; exemplifies how we did the interpreatation recently. https://journals.plos.org/plosntds/article? id=10.1371/journal.pntd.0012533#references

• Since the data analysis is faulty, so does the interpretationof findings, hence it would be impossible to proceed with the review,

• Also authors should kindly perform adjusted OR, to help control cofounders.

Reviewer #2: The manuscript demonstrates strong writing; however, to enhance its impact, the study's objectives must be explicitly defined. The discussion section needs to be more effectively structured, ensuring that comparisons with existing research are clear and compelling. It is also essential to emphasize any new knowledge or innovations that the study contributes

**********

PLOS authors have the option to publish the peer review history of their article (what does this mean?). If published, this will include your full peer review and any attached files.

If you choose “no”, your identity will remain anonymous but your review may still be made public.

**Do you want your identity to be public for this peer review?**  For information about this choice, including consent withdrawal, please see our Privacy Policy.

Reviewer #1: **Yes:** Hammed Mogaji

Reviewer #2: **Yes:** Obiageli Josephine Nebe

[NOTE: If reviewer comments were submitted as an attachment file, they will be attached to this email and accessible via the submission site. Please log into your account, locate the manuscript record, and check for the action link "View Attachments". If this link does not appear, there are no attachment files.]

**Figure resubmission:**

While revising your submission, we strongly recommend that you use PLOS’s NAAS tool (https://ngplosjournals.pagemajik.ai/artanalysis) to test your figure files. NAAS can convert your figure files to the TIFF file type and meet basic requirements (such as print size, resolution), or provide you with a report on issues that do not meet our requirements and that NAAS cannot fix.

After uploading your figures to PLOS’s NAAS tool - https://ngplosjournals.pagemajik.ai/artanalysis, NAAS will process the files provided and display the results in the "Uploaded Files" section of the page as the processing is complete. If the uploaded figures meet our requirements (or NAAS is able to fix the files to meet our requirements), the figure will be marked as "fixed" above. If NAAS is unable to fix the files, a red "failed" label will appear above. When NAAS has confirmed that the figure files meet our requirements, please download the file via the download option, and include these NAAS processed figure files when submitting your revised manuscript.

**Reproducibility:**

To enhance the reproducibility of your results, we recommend that authors of applicable studies deposit laboratory protocols in protocols.io, where a protocol can be assigned its own identifier (DOI) such that it can be cited independently in the future. Additionally, PLOS ONE offers an option to publish peer-reviewed clinical study protocols. Read more information on sharing protocols at https://plos.org/protocols?utm_medium=editorial-email&utm_source=authorletters&utm_campaign=protocols

Attachment Submitted filename: PNTD-D-26-00512 Manucript PLOS March 2026.pdf

# Author comment: Author response to Decision Letter 1

**DOI:** 10.1371/journal.pntd.0014603.r002

8 Jun 2026

Attachment Submitted filename: Response to Reviewers Comments.docx

# Peer review: Decision Letter 1

**DOI:** 10.1371/journal.pntd.0014603.r003

**Authors:** Francesca Tamarozzi

2 Jul 2026

PNTD-D-26-00512R1

Impact Assessment of Effective Rounds of Mass Drug Administration against Schistosomiasis and Soil Transmitted Helminthiasis among School Age Children in Akwa Ibom State, Nigeria

PLOS Neglected Tropical Diseases

Dear Dr. Yaro,

Thank you for submitting your manuscript to PLOS Neglected Tropical Diseases. After careful consideration, we feel that it has merit but does not fully meet PLOS Neglected Tropical Diseases's publication criteria as it currently stands. Therefore, we invite you to submit a revised version of the manuscript that addresses the points raised during the review process. It is important the points in the summary below AND highlighted in the attached copies of the manuscript are addressed satisfactorily. Please make sure that claims and figures in the text, when referring to official WHO definitions or thresholds, are complied with.

Please submit your revised manuscript by Aug 31 2026 11:59PM. If you will need more time than this to complete your revisions, please reply to this message or contact the journal office at plosntds@plos.org. When you're ready to submit your revision, log on to https://www.editorialmanager.com/pntd/ and select the 'Submissions Needing Revision' folder to locate your manuscript file.

Please include the following items when submitting your revised manuscript:

* A letter that responds to each point raised by the editor and reviewer(s). You should upload this letter as a separate file labeled 'Response to Reviewers'. This file does not need to include responses to any formatting updates and technical items listed in the 'Journal Requirements' section below.

* A marked-up copy of your manuscript that highlights changes made to the original version. You should upload this as a separate file labeled 'Revised Manuscript with Track Changes'.

* An unmarked version of your revised paper without tracked changes. You should upload this as a separate file labeled 'Manuscript'.

If you would like to make changes to your financial disclosure, competing interests statement, or data availability statement, please make these updates within the submission form at the time of resubmission. Guidelines for resubmitting your figure files are available below the reviewer comments at the end of this letter.

As the corresponding author, your ORCID iD is verified in the submission system and will appear in the published article. PLOS supports the use of ORCID, and we encourage all coauthors to register for an ORCID iD and use it as well. Please encourage your coauthors to verify their ORCID iD within the submission system before final acceptance, as unverified ORCID iDs will not appear in the published article. Only the individual author can complete the verification step; PLOS staff cannot verify ORCID iDs on behalf of authors.

We look forward to receiving your revised manuscript.

Kind regards,

Francesca Tamarozzi

Section Editor

PLOS Neglected Tropical Diseases

Francesca Tamarozzi

Section Editor

PLOS Neglected Tropical Diseases

Shaden Kamhawi

co-Editor-in-Chief

PLOS Neglected Tropical Diseases

orcid.org/0000-0003-4304-636XX

Paul Brindley

co-Editor-in-Chief

PLOS Neglected Tropical Diseases

orcid.org/0000-0003-1765-0002

**Additional Editor Comments (if provided):**

**Journal Requirements:**

If the reviewer comments include a recommendation to cite specific previously published works, please review and evaluate these publications to determine whether they are relevant and should be cited. There is no requirement to cite these works unless the editor has indicated otherwise.

1) Please ensure that the CRediT author contributions listed for every co-author are completed accurately and in full.

At this stage, the following Authors/Authors require contributions: Kenneth Nnamdi Opara, Clement Ameh Yaro, Nnamso Williams Ikpeida, Aliyu Mohammed, Hadiza Joy Marcus, Kingsley Azu, Salisu Ahmad, Ellie Leaning, Moses Aderogba, Louise-Makau Barasa, Antia Etop, Imeh Sylvester Udoh, Fatai Oyediran, Imabong O. Umah, Solomon M. Jacob, Gideon U. Ntuen, Veronica S. Augustine, and Halima Toro. Please ensure that the full contributions of each author are acknowledged in the "Add/Edit/Remove Authors" section of our submission form.

The list of CRediT author contributions may be found here: https://journals.plos.org/plosntds/s/authorship#loc-author-contributions

2) Please upload all main figures as separate Figure files in .tif or .eps format. For more information about how to convert and format your figure files please see our guidelines:

https://journals.plos.org/plosntds/s/figures

3) We have noticed that you have uploaded Supporting Information files, but you have not included a list of legends. Please add a full list of legends for your Supporting Information files after the references list.

**Reviewers' comments:**

Reviewer's Responses to Questions

**Key Review Criteria Required for Acceptance?**

As you describe the new analyses required for acceptance, please consider the following:

**Methods**

-Are the objectives of the study clearly articulated with a clear testable hypothesis stated?

-Is the study design appropriate to address the stated objectives?

-Is the population clearly described and appropriate for the hypothesis being tested?

-Is the sample size sufficient to ensure adequate power to address the hypothesis being tested?

-Were correct statistical analysis used to support conclusions?

-Are there concerns about ethical or regulatory requirements being met?

Reviewer #1: T

Reviewer #2: The objectives were well articulated, with appropriate study design. The study population- SAC was clearly described and appropriate for the hypothesis that were tested. The study has a robust sample size. Correct statistical analysis were deployed and that supported the conclusions that emanated from the study. No concern whatsoever.

**********

**Results**

-Does the analysis presented match the analysis plan?

-Are the results clearly and completely presented?

-Are the figures (Tables, Images) of sufficient quality for clarity?

Reviewer #1: (No Response)

Reviewer #2: The analysis matched the data plan and the results clearly presented. Further statistical analysis were done, especially in respect of OR and AOR and other statistical variables. The figures and tables are of sufficient quality

**********

**Conclusions**

-Are the conclusions supported by the data presented?

-Are the limitations of analysis clearly described?

-Do the authors discuss how these data can be helpful to advance our understanding of the topic under study?

-Is public health relevance addressed?

Reviewer #1: (No Response)

Reviewer #2: The conclusions are supported by the data presented. The limitations were described. The authors discussed how the data could be helpful in advancing our understanding of the subject matter. The public health relevance was described, especially the programmatic interpretation of the implications of the findings to the Akwa Ibom State project,

**********

**Editorial and Data Presentation Modifications?**

Use this section for editorial suggestions as well as relatively minor modifications of existing data that would enhance clarity. If the only modifications needed are minor and/or editorial, you may wish to recommend “Minor Revision” or “Accept”.

Reviewer #1: (No Response)

Reviewer #2: The manuscript looks good and should be accepted after "Minor Revision". There are few areas of some concerns that the authors should address as highlighted in the manuscript.

**********

**Summary and General Comments**

Use this section to provide overall comments, discuss strengths/weaknesses of the study, novelty, significance, general execution and scholarship. You may also include additional comments for the author, including concerns about dual publication, research ethics, or publication ethics. If requesting major revision, please articulate the new experiments that are needed.

Reviewer #1: • The authors have satisfactorily addressed most of the comments but the most important one regarding the programmatic interpretation of the data is not revised. They have interpreted their data based on old thresholds used before initiating MDA i.e., 0-19.9; 20-49.9 and >50. However, the WHO thresholds for LGA that have had effective MDA for more than 5 years is < 2%, ≥ 2% to < 10%, ≥ 10 % to < 20%, and ≥ 20%. So most of the tables and figures presented needs to be revised accordingly.

• Also, for the intensity. It is unclear how authors interpreted the intensity presented on table 6, each of the species has different egg count for intensity threshold. Hence, authors are expected to present intensity per STH species based on that egg count. Please see ; The intensity of Ascaris lumbricoides was classified as low (1–4,999 EPG), moderate (5,000–49,999 EPG), or high (≥50,000 EPG) For hookworms, intensities were classified as low (1–1,999 EPG), moderate (2,000–3,999 EPG), or high (≥4,000 EPG) Trichuris trichiura infections were categorized as low (1–999 EPG), moderate (1,000–9,999 EPG), or high (≥10,000 EPG) [15].

Please see a copy of a similar manuscript published; https://www.mdpi.com/2414-6366/11/1/24

Reviewer #2: The manuscript is well written, with robust statistical analysis. However the authors should address few comments and observations made on the manuscript.

The authors should cross-check the no of LGAs being considered not requiring PC. The actually number is 9 not 11 as reported. Ibiono Ibom which recorded 19.91% should not be overlooked or under classified, but approximated to 20%.

The claim regarding the potential elimination of schistosomiasis in Akwa Ibom State is fundamentally misleading; the state is not endemic for schistosomiasis based on baseline findings. Moreover, any assertion of elimination must be substantiated through a comprehensive evaluation that adheres to the criteria and assessment procedures established by the World Health Organization (WHO). This evaluation must include thorough assessments of humans, snails, One Health initiatives, and Water, Sanitation, and Hygiene (WASH) interventions. Credible data must be generated and submitted to the WHO, along with preparations for a dossier submission for validation and certification, before making definitive claims about elimination.

**********

PLOS authors have the option to publish the peer review history of their article (what does this mean?). If published, this will include your full peer review and any attached files.

If you choose “no”, your identity will remain anonymous but your review may still be made public.

**Do you want your identity to be public for this peer review?**  For information about this choice, including consent withdrawal, please see our Privacy Policy.

Reviewer #1: **Yes:** Hammed Mogaji

Reviewer #2: **Yes:** Obiageli Josephine Nebe

[NOTE: If reviewer comments were submitted as an attachment file, they will be attached to this email and accessible via the submission site. Please log into your account, locate the manuscript record, and check for the action link "View Attachments". If this link does not appear, there are no attachment files.]

**Figure resubmission:**

While revising your submission, we strongly recommend that you use PLOS’s NAAS tool (https://ngplosjournals.pagemajik.ai/artanalysis) to test your figure files. NAAS can convert your figure files to the TIFF file type and meet basic requirements (such as print size, resolution), or provide you with a report on issues that do not meet our requirements and that NAAS cannot fix.

After uploading your figures to PLOS’s NAAS tool - https://ngplosjournals.pagemajik.ai/artanalysis, NAAS will process the files provided and display the results in the "Uploaded Files" section of the page as the processing is complete. If the uploaded figures meet our requirements (or NAAS is able to fix the files to meet our requirements), the figure will be marked as "fixed" above. If NAAS is unable to fix the files, a red "failed" label will appear above. When NAAS has confirmed that the figure files meet our requirements, please download the file via the download option, and include these NAAS processed figure files when submitting your revised manuscript.

**Reproducibility:**

To enhance the reproducibility of your results, we recommend that authors of applicable studies deposit laboratory protocols in protocols.io, where a protocol can be assigned its own identifier (DOI) such that it can be cited independently in the future. Additionally, PLOS ONE offers an option to publish peer-reviewed clinical study protocols. Read more information on sharing protocols at https://plos.org/protocols?utm_medium=editorial-email&utm_source=authorletters&utm_campaign=protocols

Attachment Submitted filename: PNTD-D-26-00512_R1 (2) June 2026.pdf

# Author comment: Author response to Decision Letter 2

**DOI:** 10.1371/journal.pntd.0014603.r004

20 Jul 2026

Attachment Submitted filename: Response_to_Reviewers_Comments_auresp_2.docx

# Editor report: Decision Letter 2

**DOI:** 10.1371/journal.pntd.0014603.r005

**Authors:** Peter Fischer

21 Jul 2026

Dear Dr. Yaro,

We are pleased to inform you that your manuscript 'Impact Assessment of Effective Rounds of Mass Drug Administration against Schistosomiasis and Soil Transmitted Helminthiasis among School Age Children in Akwa Ibom State, Nigeria' has been provisionally accepted for publication in PLOS Neglected Tropical Diseases.

Before your manuscript can be formally accepted you will need to complete some formatting changes, which you will receive in a follow up email. A member of our team will be in touch with a set of requests.

Please note that your manuscript will not be scheduled for publication until you have made the required changes, so a swift response is appreciated.

IMPORTANT: The editorial review process is now complete. PLOS will only permit corrections to spelling, formatting or significant scientific errors from this point onwards. Requests for major changes, or any which affect the scientific understanding of your work, will cause delays to the publication date of your manuscript.

Should you, your institution's press office or the journal office choose to press release your paper, you will automatically be opted out of early publication. We ask that you notify us now if you or your institution is planning to press release the article. All press must be co-ordinated with PLOS.

Thank you again for supporting Open Access publishing; we are looking forward to publishing your work in PLOS Neglected Tropical Diseases.

Best regards,

Peter U Fischer

Section Editor

PLOS Neglected Tropical Diseases

Francesca Tamarozzi

Section Editor

PLOS Neglected Tropical Diseases

Shaden Kamhawi

co-Editor-in-Chief

PLOS Neglected Tropical Diseases

orcid.org/0000-0003-4304-636XX

Paul Brindley

co-Editor-in-Chief

PLOS Neglected Tropical Diseases

orcid.org/0000-0003-1765-0002

***********************************************************

p.p1 {margin: 0.0px 0.0px 0.0px 0.0px; line-height: 16.0px; font: 14.0px Arial; color: #323333; -webkit-text-stroke: #323333}span.s1 {font-kerning: none

# Editor report: Acceptance letter

**DOI:** 10.1371/journal.pntd.0014603.r006

**Authors:** Peter Fischer

Dear Dr. Yaro,

We are delighted to inform you that your manuscript, "Impact Assessment of Effective Rounds of Mass Drug Administration against Schistosomiasis and Soil Transmitted Helminthiasis among School Age Children in Akwa Ibom State, Nigeria," has been formally accepted for publication in PLOS Neglected Tropical Diseases.

We have now passed your article onto the PLOS Production Department who will complete the rest of the publication process. All authors will receive a confirmation email upon publication.

The corresponding author will soon be receiving a typeset proof for review, to ensure errors have not been introduced during production. Please review the PDF proof of your manuscript carefully, as this is the last chance to correct any scientific or type-setting errors. Please note that major changes, or those which affect the scientific understanding of the work, will likely cause delays to the publication date of your manuscript. Note: Proofs for Front Matter articles (Editorial, Viewpoint, Symposium, Review, etc...) are generated on a different schedule and may not be made available as quickly.

Soon after your final files are uploaded, the early version of your manuscript will be published online unless you opted out of this process. The date of the early version will be your article's publication date. The final article will be published to the same URL, and all versions of the paper will be accessible to readers.

For Research Articles, you will receive an invoice from PLOS for your publication fee after your manuscript has reached the completed accept phase. If you receive an email requesting payment before acceptance or for any other service, this may be a phishing scheme. Learn how to identify phishing emails and protect your accounts at https://explore.plos.org/phishing.

Thank you again for supporting open-access publishing; we are looking forward to publishing your work in PLOS Neglected Tropical Diseases.

Best regards,

Shaden Kamhawi

co-Editor-in-Chief

PLOS Neglected Tropical Diseases

Paul Brindley

co-Editor-in-Chief

PLOS Neglected Tropical Diseases
